# Supplementary material for: Effects of spatial smoothing on group-level differences in functional brain networks
Source: Netw Neurosci. 2020 Jul 1;4(3):556–74. doi: 10.1162/netn_a_00132 (PMC7462426; doi:10.1162/netn_a_00132)
Supplement: Supplementary file 1 [file netn-04-556-s001.pdf]

Triana, A. M., Glerean, E., Saramäki, J., & Korhonen, O. (2020). Supporting information for "Effects of spatial smoothing on grouplevel differences in functional brain networks." *Network Neuroscience*, *4*(3), 556–574. https://doi.org/10.1162/netn_a_00132

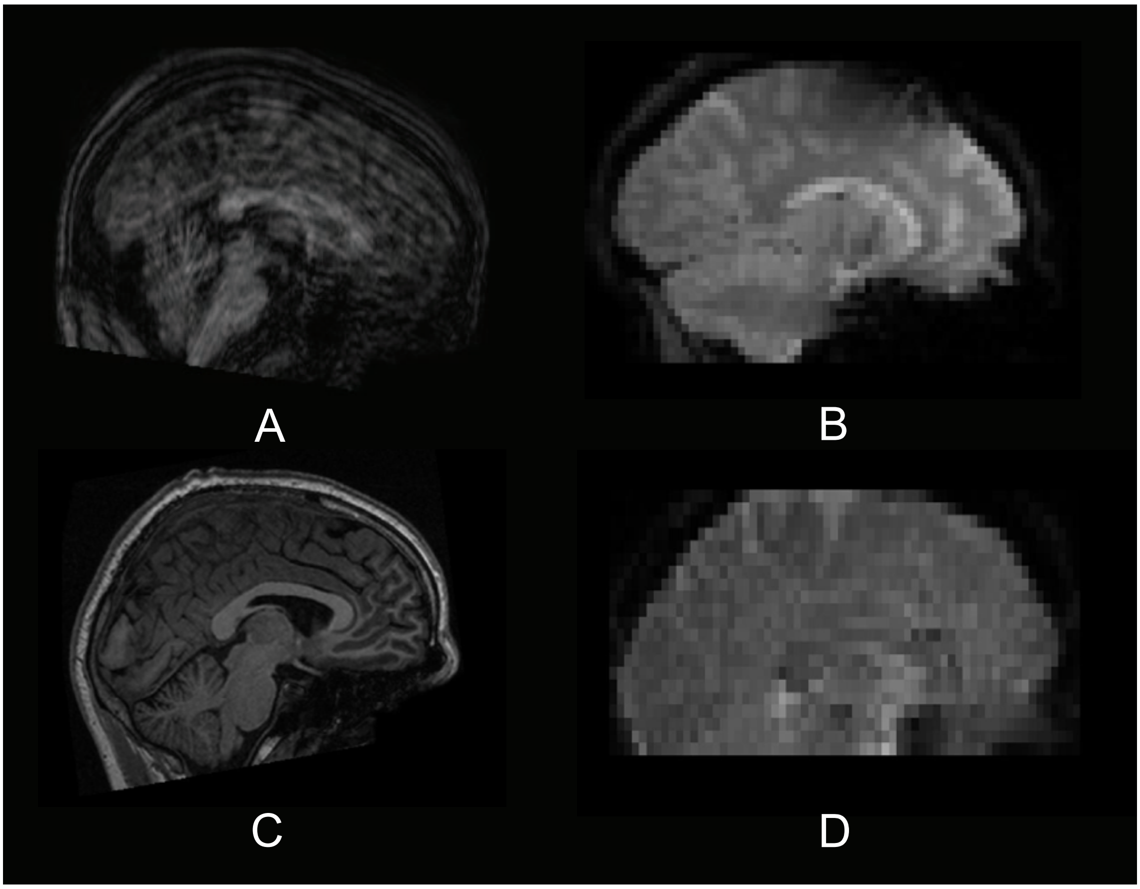

Figure A1: MRI artifact examples. These images present some of the artifacts found in the subjects from ABIDE. Notes about the artifacts were made in supplementary table 2. (A) motion in the structural MRI, (B) unexplained changes in intensity, (C) distortion from metallic objects, (D)brain parts missing.

Image quality control

The unpreprocessed structural and fMRI data (as downloaded from `http://fcon_1000.projects.nitrc.org/indi/abide/`) were visually inspected. Different artifacts were found in the structural MRIs, e.g. missing brain parts, motion, distortions due to hair products, etc. Example images of these findings are shown in supplementary file A1. Notes of these artifacts were taken, as seen in the supplementary table 2. Afterward, the subjects' fMRIs were visually inspected. Careful attention was put into detecting motion and its severity: if the subject moved during most of the scan and the movement was perceptible at this stage, the subject was immediately discarded, for example subject 0050483. Other found artifacts include missing brain sections and regions with unexplained changes in intensity or black voxels. Subjects were discarded if the artifacts interfered with the subsequent preprocessing steps, for example, all subjects with missing brain regions were immediately discarded.

After the image quality control, we preprocessed the fMRI data as described in the Materials and Methods section of the main article. Then, additional subjects were discarded because of the framewise displacement criteria also described in the the Materials and Methods section of the main article.

Weighted, thresholded networks

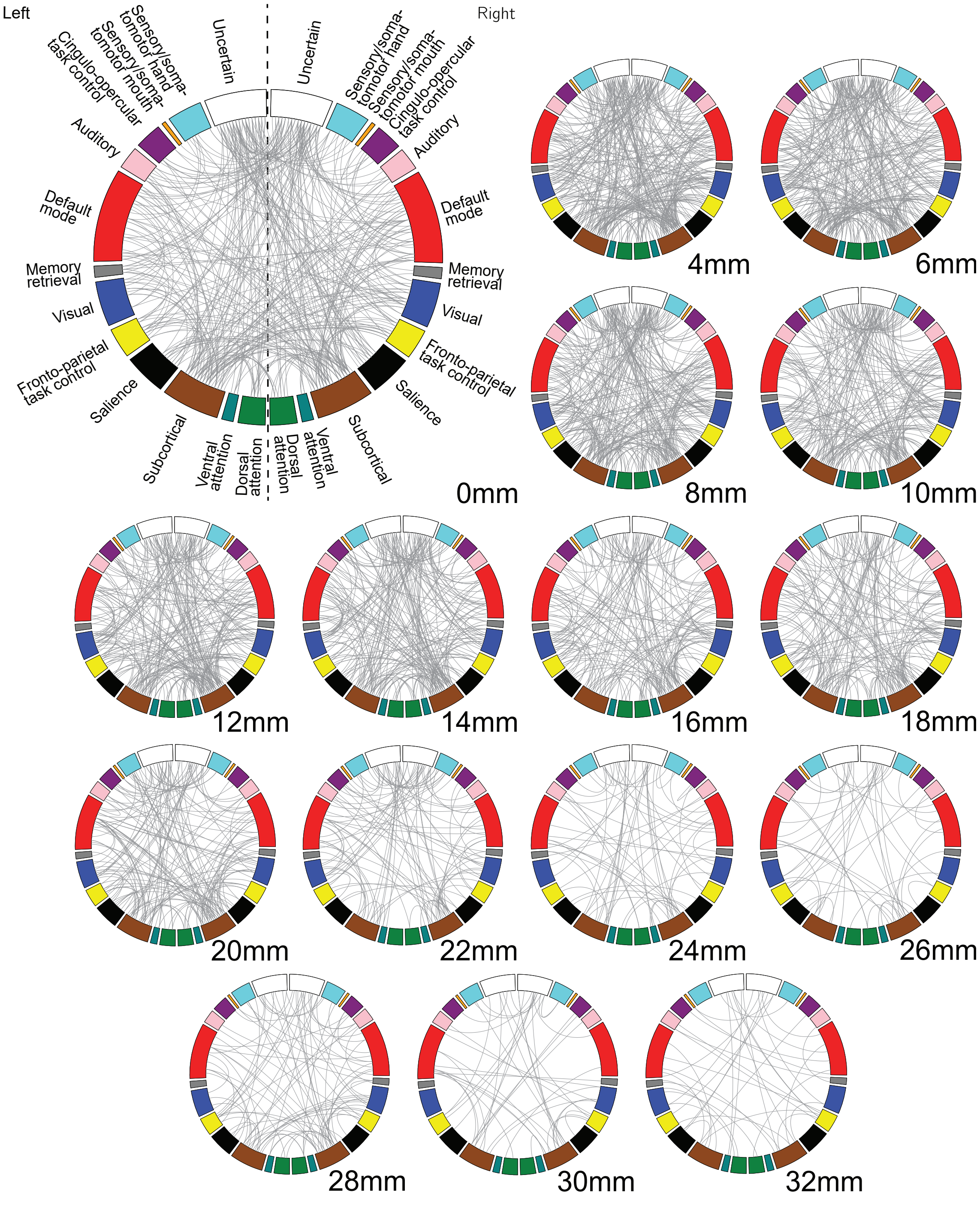

Figure A2: Group-level differences in thresholded, weighted, resting-state functional networks according to the smoothing kernel (ABIDE dataset, Brainnetome parcellation). The circos plots show the between-groups connectivity differences identified by permutation tests as described in the section Network comparison of the main article. The nodes are grouped into systems according to Power et al. (2011), colored accordingly, and split in left and right hemispheres. The choice of smoothing kernel changes the detected connectivity differences. The connections found at low kernels differ drastically from those at high kernels in terms of comprised links. A decrease of subnetwork density is evidenced as the smoothing kernel increases.

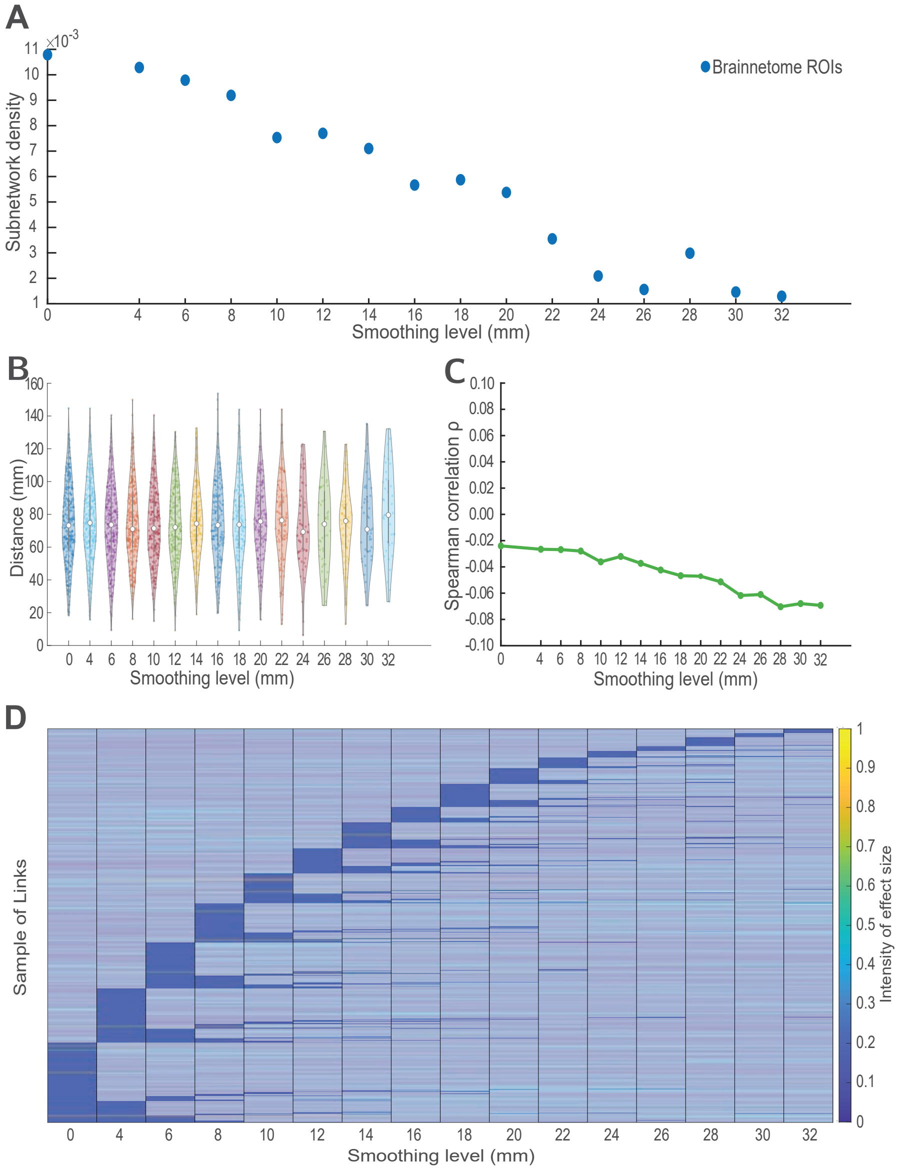

Figure A3: Characteristics of between-group differences according to the smoothing kernel for thresholded, weighted networks (ABIDE dataset, Brainnetome parcellation). Based on the network differences identified by the permutation tests, we see that: (A) The choice of smoothing kernel affects the number of significantly different links. The number of significantly different links decreases as the smoothing kernel increases. (B) Spatial smoothing alters the distance profile of detected links with no clear pattern. (C) Larger kernels are associated with a decrease in the detection of longer links. At first sight, a decline in the value of ρ with increasing FWHM highlights a stronger negative correlation between the length of the links and their T-statistic as the smoothing kernel increases. In other words, using larger kernels decreases the chance of finding long links. (D) Effect sizes of the significantly different links change depending on the smoothing kernel. The y-axis shows the links which permutation tests yielded to be different between groups at some kernel. The links (y-axis) are organized according to the smallest kernel in which they are detected and the number of smoothing levels in which they appear statistically significant (corrected p-value α<0.05); for example, all links which are significant at 0 mm are shown at the bottom of the plot, then on top of them, we show all links which are significant at 4mm, but are not significant at 0mm. This organization follows until all smoothing levels are shown. The plot highlights those kernels at which the links are found significant. No links are detected at all smoothing levels. Conversely, some links are only observed when a particular kernel is used. Moreover, some links are detected at kernels that are not consecutive, evidencing the unpredictability of the effects. In general, the effect sizes are small.

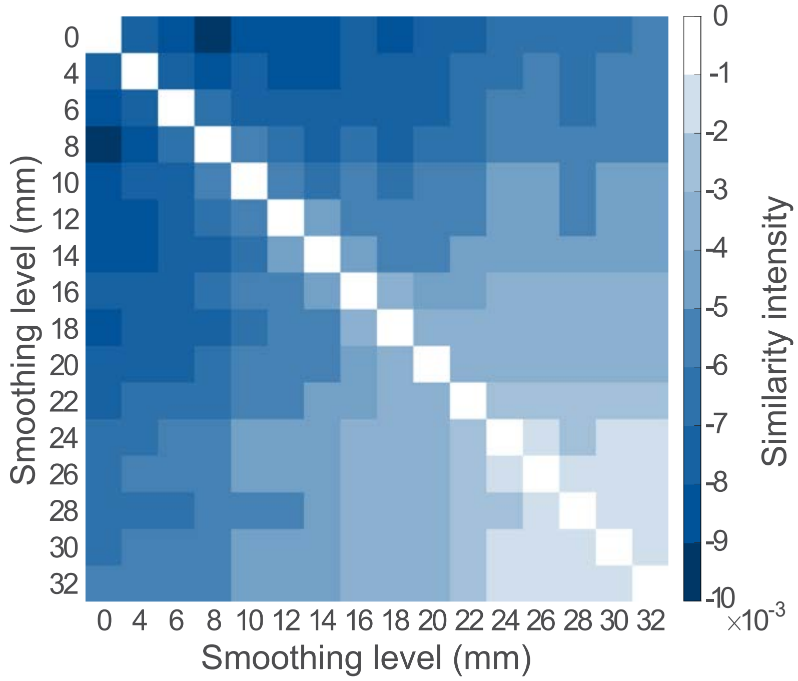

Figure A4: Similarity matrix between the identified group-level network differences at each smoothing level for thresholded, weighted matrices for the ABIDE dataset. Brighter colors represent less differences in the structure of the links. Surprisingly, subnetwork pair 0-8 mm exhibit the largest difference, even larger than e.g. subnetwork pair 0-30 mm. This evidences the variation in the detected links.

Generality of the results

Next, we investigate whether results discussed in the main article are robust against changes in the analysis pipeline: different NBS suprathresholds, parcellations, density thresholds for thresholded networks, datasets, and spatial smoothing methods.

Results hold for different NBS suprathresholds

The different amounts of significantly different links for different kernels might also be explained by non-linearities in the method used to compare the groups (NBS). Two parameters of NBS may affect the links comprising the subnetwork: i) the suprathreshold level that defines the F-statistic threshold for individual links and ii) the significance criterion that defines the significance of the connected component or subnetwork. Because spatial smoothing influences the effect size of the individual links in an irregular manner, some links may cross the suprathreshold and be discarded from the subnetwork, even if the effects of smoothing are small. To investigate the effect of NBS suprathreshold, we run our analysis for 8 different F-statistic suprathresholds (2.25, 4, 6.25, 9, 12.25, 16, 20.25, and 25). As expected, smaller suprathresholds yield denser subnetworks (Fig. A5 A). For suprathresholds 2.25, 4, 6.25, 9, and 12.25, the subnetwork density increases with the smoothing kernel: larger kernel widths yield more differences in the group comparison. This is expected as lower suprathresholds cause more links to be included in the largest connected component. However, for larger suprathresholds (16, 20.25, and 25), the effect is different from the results reported above (Fig. A5 B). In particular, for suprathreshold 16, the subnetwork density first increases and then decreases. Since increased spatial smoothing makes the image more averaged, we hypothesize that there is an inflexion point, where an increase of smoothing causes a decrease in the number of links comprising the connected component until no further differences are detected. Based on Fig. A5, we hypothesize that for smaller suprathresholds, such inflexion point would be achieved at kernels larger than FWHM = 32 mm. However, this hypothesis remains untested, mainly because in practical terms, kernels larger than 12 mm are rarely used.

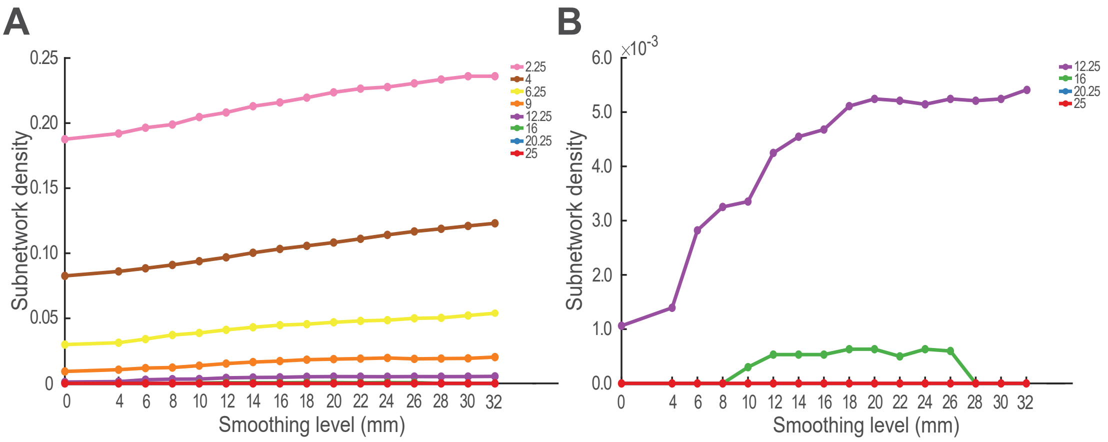

Figure A5: NBS suprathresholds influence the number of significantly different links comprising the subnetwork for the ABIDE dataset. (A) We compare the number of links yielded by NBS at 8 different suprathreshold values. As expected, smaller suprathresholds yield denser networks as more links are selected, making the connected component larger. For these suprathresholds, larger smoothing kernels yield denser subnetworks. (B) Larger suprathresholds have different effects to the reported in the Results section of the main article. No differences are found for suprathreshold = 20.25 and 25.

Results hold for different parcellations

To make sure that our results are not an artefact that can be attributed to the specific parcellation used, we repeat our analysis using the Craddock atlas Craddock et al. (2012): Craddock100 (Supplementary Fig. A6) and Craddock350 (Supplementary Fig. A7). This allows us to evaluate the effects of spatial smoothing in parcellations that are sparser and denser than Brainnetome.

In general, we observe for the Craddock parcellations similar results as for Brainnetome. Connectivity between subcortical (SUB) and sensorimotor hand (SMH) and cingulo-opercular areas differs between groups in all parcellations (Fig. 2 of the main article and Figs. A6 and A7). Similarly as for the Brainnetome parcellation, the NBS subnetwork density increases with the smoothing kernel FWHM (Fig. A8) and link effect sizes vary with smoothing on a non-systematic way (Fig. A9). Likewise, subnetworks are most similar when smoothing kernel widths are only 2 or 4 mm apart (Fig. A10). In the Craddock parcellations, the relationship between the smoothing kernel FWHM and the physical length of the subnetwork links is similar to Brainnetome although less strong (Fig. A11). Further, the negative correlation that we observe between the link effect size and smoothing kernel FWHM is similar to Brainnetome.

The observed trends are less clear for the Craddock100 parcellation. This is mainly because the subnetworks observed at the fixed suprathresholds of 12.25 are small and some of the links of these subnetworks are detected at all smoothing levels. It is possible that for the ABIDE dataset, the Craddock100 number of ROIs reflect the average level of activity in larger functional modules, rather than in functional areas.

We also repeat our analysis for thresholded, weighted networks using the Craddock atlases (Figs. A12 and A13). Trends remain unchanged for the density of the subnetworks (Fig. A14) and effect size (Fig. A15). The trends in the length profiles of the subnetwork links differ between parcellations (Fig. A16 A and B). Nevertheless, the relationship between link length and smoothing kernel FWHM for Craddock350 is similar as for Brainnetome, while Craddock100 differs from the other parcellations (Fig. A16 C). Conversely, the similarity of subnetworks obtained at different smoothing levels follows in Craddock100 the same pattern as in Brainnetome, while in Craddock350 the subnetworks observed at small smoothing kernels (0, 4, and 6 mm) are notably different from the subnetworks observed at other kernels (Fig. A17).

Network density influences comparison of thresholded networks

The selected network density may affect the group comparison in thresholded, weighted networks. To investigate its possible effects in our results, we compare weighted networks thresholded at different densities (5, 7, 9, 10, 11, 13, 15, 17, 19 and 20 %). We find that the choice of network density highly affects the results (Fig. A18). For the lower densities (5 and 7 % in Fig. A18 A), an increment in the smoothing kernel width generates a decrease in the subnetwork density. However, the other thresholds show no distinguishable pattern (Fig. A18 B). In summary, observed differences in network structure depend highly on the network density.

Finally, the lack of significant between-group differences in some graph measures (degree and mean clustering coefficient) may be related to network density. Thus, we compare binary networks thresholded at different densities (5, 7, 9, 10, 11, 13, 15, 17, 19 and 20 %) in terms of the graph measures described in the section Network comparison. We do not find any significant between-group differences in degree or the mean clustering coefficient at any threshold. Although we detect differences in betweenness centrality (Supplementary Table 8) and clustering coefficient (Supplementary Table 9), few of these differences have any consistency across different thresholds. The global efficiency (Supplementary Table 10) differs consistently between groups across different thresholds. However, these differences are detected only at smoothing levels FWHM$\geq$24 mm. Similarly, other nodes differ in local efficiency (Supplementary Table 11). However, only nodes 91 (left extreme lateroventral area37), 100 (right caudolateral of area 20), and 173 (left dorsal dysgranular insula) are detected for commonly used kernels (FWHM$\leq$12 mm). In general, statistically significant results appear sporadically and with no pattern.

Results hold for other datasets

To consider the generalizability of our results across datasets, we repeat the analysis for a second, independent dataset (UCLA) using the Brainnetome parcellation. As the patients of the UCLA dataset suffer from the bipolar disorder instead of ASD, the detected subnetworks differ structurally from those of ABIDE (Fig. A19). Nevertheless, an increase in the smoothing kernel causes an increment in the number of links in the subnetwork similarly as in the ABIDE data (Fig. A20 A). These results remain unchanged even when the size of the ROIs is fixed (Fig. A20 A). The trends in the effect size (Fig. A20 D) and similarity between subnetworks (Fig. A21) detected from the UCLA data are similar to those detected from ABIDE. We observe a minimal variation in the distance profiles of the UCLA subnetworks according to the smoothing kernel (Fig. A20 B). The

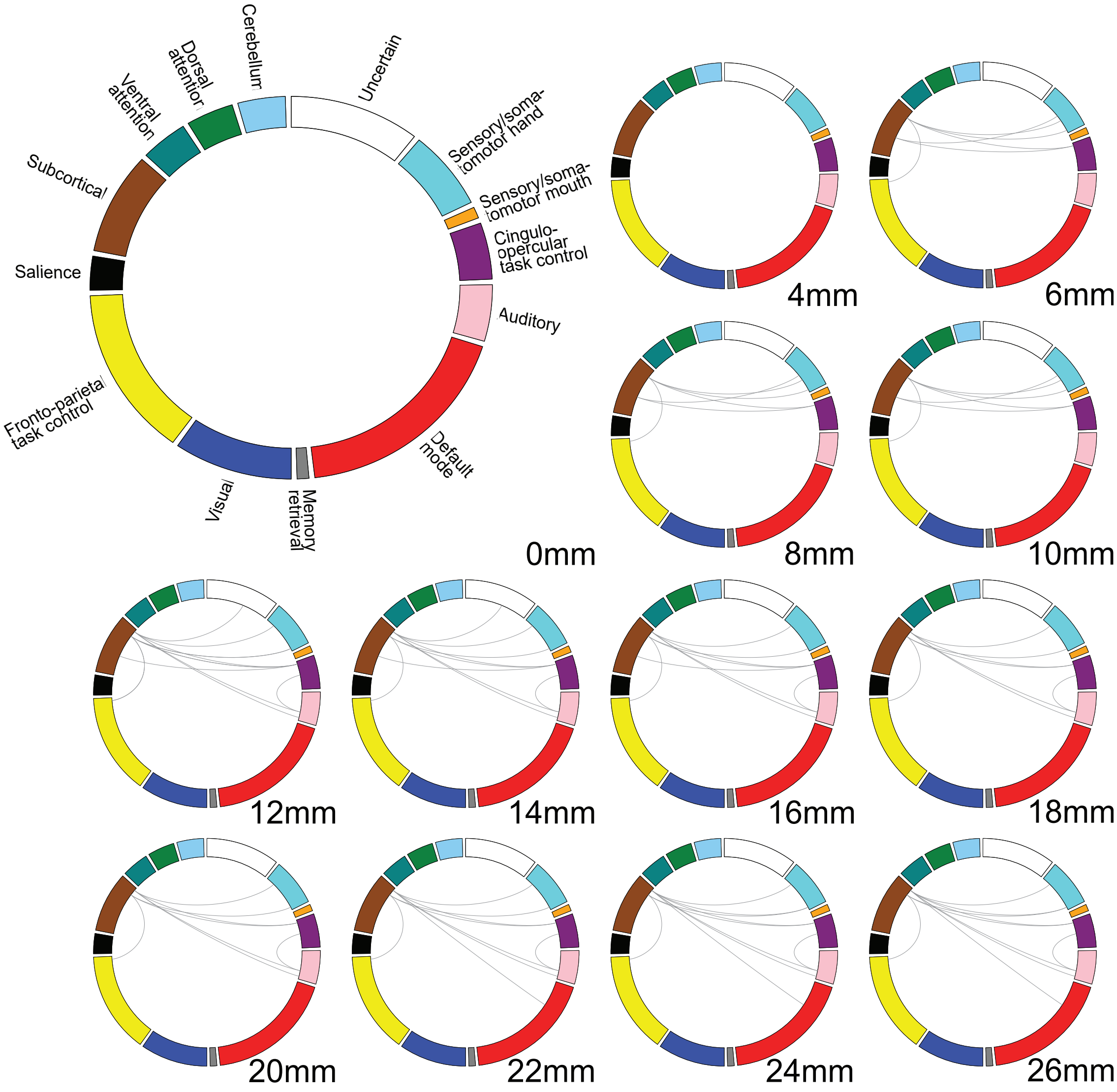

Figure A6: Group-level differences in resting-state functional networks according to the smoothing kernel (ABIDE dataset, Craddock100 parcellation). The circos plots show the between-groups connectivity differences identified by NBS as described in the section Network comparison of the main article. The nodes are grouped into systems according to Power et al. (2011) and colored accordingly. The choice of smoothing kernel affects the detection of connectivity differences. Few links are determined to be significantly different for FWHM $\leq$ 26 mm. However, link composition of the subnetworks remains consistent for many levels.

correlation analysis shows that unlike in the ABIDE data, an increase in the smoothing kernel is first associated with an increase in the probability of longer links being detected until a tipping point, at which the trend is reversed (Fig. A20 C).

The effects of the NBS suprathreshold on the results are partly the same for the UCLA and ABIDE datasets. At the larger NBS suprathresholds, the subnetwork density increases with the increasing smoothing kernel FWHM (Fig. A22). Analyzing all the suprathresholds, we see that in the UCLA dataset this increase reaches a peak around FWHM=24 mm, after which the subnetwork density decreases abruptly towards zero. As stated above, in the ABIDE data a similar inverted u-shape trend is visible for the suprathreshold 16 (Fig. A5 B). Unlike in the ABIDE data, in the UCLA dataset lower suprathresholds do not yield denser subnetworks.

The drastic effects of network density on the number of significantly different links in thresholded, weighted networks are also visible in the UCLA dataset (Fig. A23). However, the effects are more uniform than for ABIDE; for most of the tested densities, an increase in the smoothing kernel

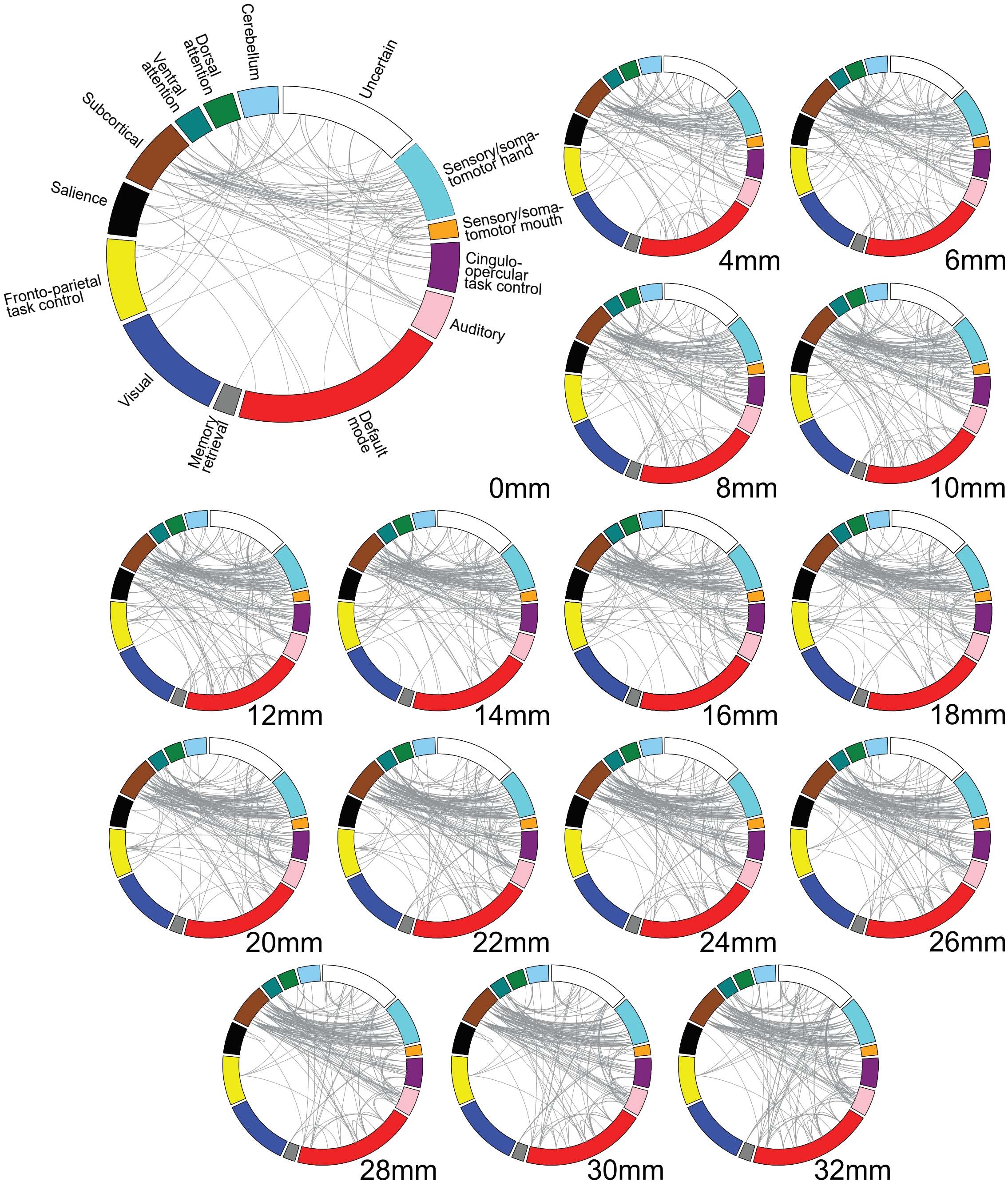

Figure A7: Group-level differences in resting-state functional networks according to the smoothing kernel (ABIDE dataset, Craddock350 parcellation). The circos plots show the between-groups connectivity differences identified by NBS as described in the section Network comparison of the main article. The nodes are grouped into systems according to Power et al. (2011) and colored accordingly. The choice of smoothing kernel affects the detection of connectivity differences, changing the structure of the yielded subnetwork. Connectivity between subcortical areas and sensory/somatomotor hand and cingulo opercular areas are evident for all smoothing levels, whereas connectivity in the other areas change.

is associated with a decrease in the number of significantly different links. Given the large number of significant links found in the UCLA data at the 7% density used for analysing the ABIDE data, we will report the results at the network density of 13%. Similarly as in the ABIDE data, the number of links is large and there is no obvious pattern in the connectivity differences (Fig. A24). Nevertheless, similarly to ABIDE, we find a decrease in the number of links with an increase of smoothing level (Fig. A25 A). Although we see variations in the link length profile (Fig. A25 B),

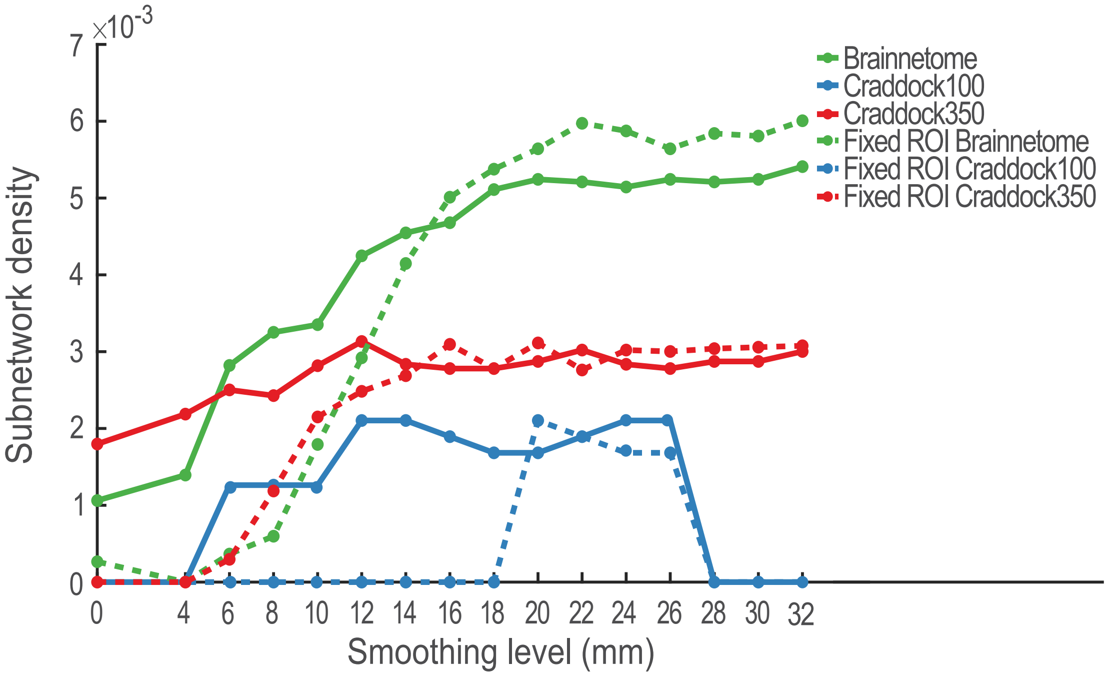

Figure A8: Variation of between-group differences according to the smoothing kernel for different parcellations (ABIDE dataset). Based on the network differences identified by NBS, we see that the choice of smoothing kernel affects the number of links of the subnetworks. The number of links in the subnetwork increases for Brainnetome and Craddock350 parcellations. This pattern is present even when the ROIs are artificially constructed as spheres of constant ratios based on the Brainnetome and Craddock350 ROI centroids. For the Craddock100 parcellation, the number of links in the subnetwork increases and then it decreases after a certain kernel (FWHM=26 mm). The pattern is also present when analyzing the data with artificial spheres of fixed ratios for the Craddock100 parcellation.

no association is found between the smoothing kernel FWHM and the correlation between link length and T-statistic, ρ (Fig. A25 C). Similarity between subnetworks (Fig. A26) and link effect size (Fig. A25 D) follow the trends found in ABIDE.

Finally, we find no differences for global efficiency and mean clustering coefficient for binary, thresholded networks constructed from the UCLA data. Similar to ABIDE, we find some significant differences for some nodes in betweenness centrality (Supplementary Table 12) and clustering coefficient (Supplementary Table 13), but such differences appear in no discernible pattern. Conversely, we find significant differences for local efficiency (Supplementary Table 14) at several network densities, most of them at large kernels. Remarkably, we find significant differences in node degree for ROIs right medial area 8 (node 2) and right caudal temporal thalamus (node 244) at commonly used kernels (FWHM$\leq$12). These differences are consistent across thresholds and for most of the commonly used kernels (Supplementary Fig. A27 and Supplementary Table 15).

Results hold when intrinsic smoothness is considered

Finally, we repeated the analysis of both datasets using the adaptive smoothing method (see the Methods section) using the Brainnetome parcellation. Results obtained using adaptive smoothing are similar to the previously reported. The observed NBS subnetwork structure observed with the non-adaptive Gaussian smoothing at most kernel sizes resembles the main results (Fig. 2 of the main article vs. Fig. A29 for the ABIDE dataset, and Fig. A19 vs. Fig. A37 for the UCLA dataset). However, at low kernel sizes (FWHM$\leq$10 mm), the links seen between subcortical areas (SUB) and sensory/somatomotor hand areas (SSH) in the ABIDE data are not present when adaptive smoothing is used. Instead, the connections between subcortical areas, the default-mode network, sensory/somatomotor hand areas, auditory, and cingulo-opercular task control areas found at FWHM=0 mm are also yielded at FWHM=4 mm and FWHM=6 mm. For the UCLA

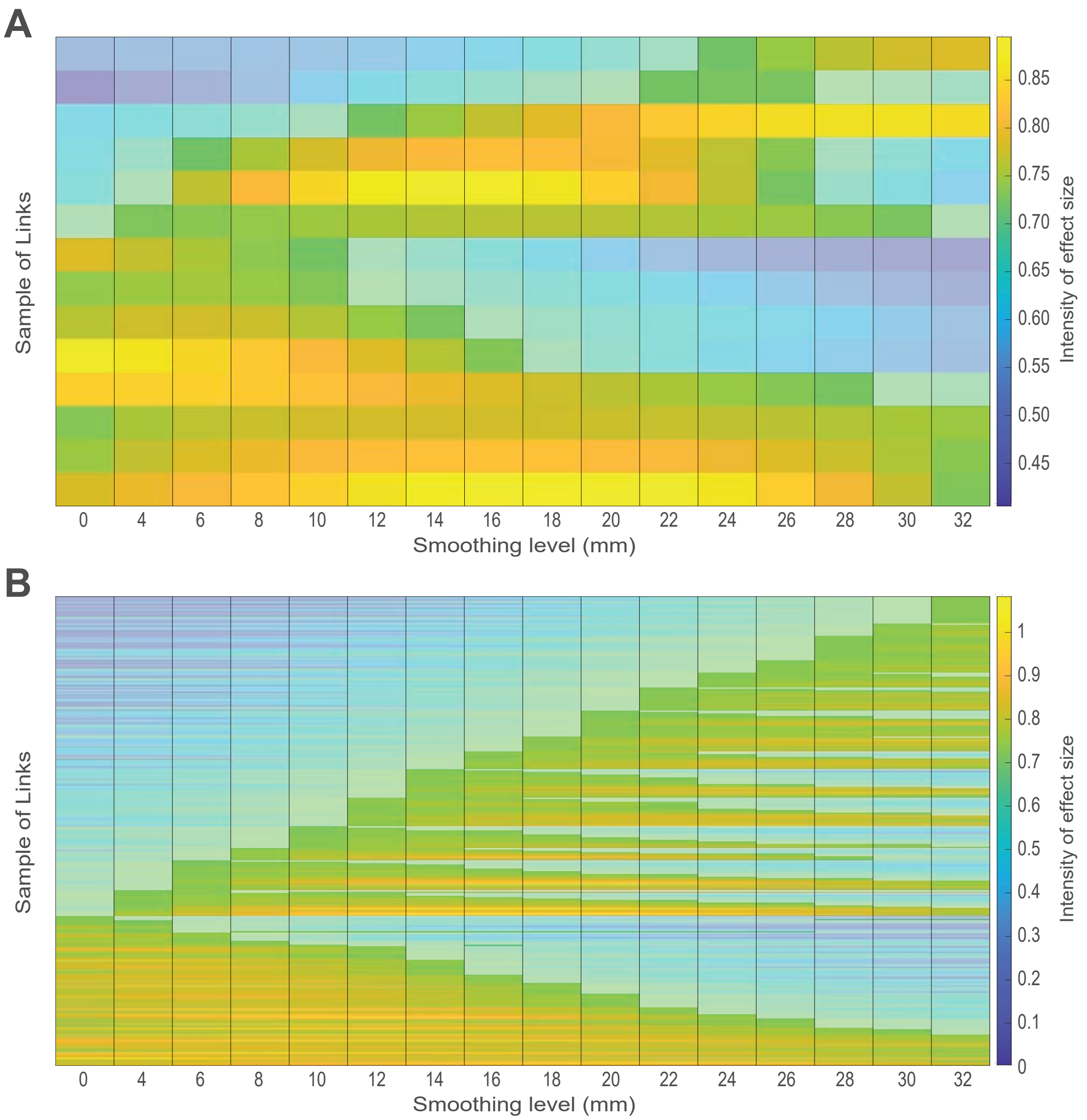

Figure A9: Effect-size variation in the between-group differences according to the smoothing kernel (ABIDE dataset) for the Craddock parcellations. Based on the network differences identified by the NBS, we see that the effect sizes of the significantly different links change depending on the smoothing kernel for both parcellations, (A) Craddock100 and (B) Craddock350. The figure shows the smoothing kernels on the x-axis, ordered from smallest (0 mm) to largest (32 mm). In the y-axis, the figure shows the links which NBS identified as different between groups at some kernel. The links (y-axis) are organized according to the smallest kernel in which they are detected and the number of smoothing levels in which they appear statistically significant ($\alpha<0.05$); for example, all links which are significant at 0 mm are shown at the bottom of the plot, then on top of them, we show all links which are significant at 4mm, but are not significant at 0mm. This organization follows until all smoothing levels are shown. The plot highlights those kernels at which the links are found significant. No links are detected at all smoothing levels. Conversely, some links are only observed when a particular kernel is used.

dataset, we find no differences at FWHM=4 mm with adaptive smoothing but find differences with non-adaptive Gaussian smoothing (Fig. A37). Conversely, we find differences at FWHM=8 mm and FWHM$\geq$26 mm with adaptive smoothing, where no differences are found with non-adaptive Gaussian smoothing.

Despite these discrepancies, the overall trends remain unchanged when using adaptive smoothing for all aspects analyzed in this article (Fig. 3 of the main article vs. Fig. A30 for the ABIDE

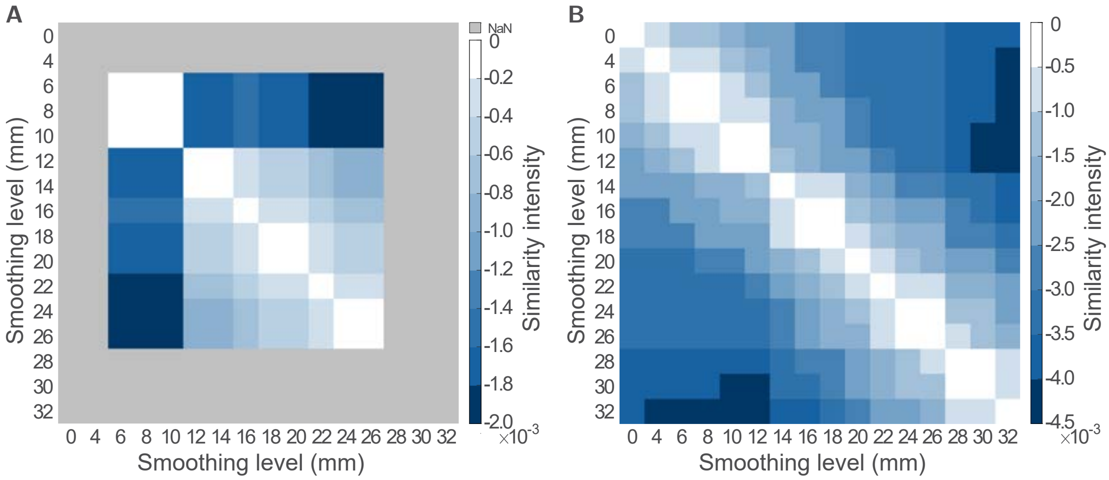

Figure A10: Similarity matrix between the identified group-level network differences (subnetworks) at each smoothing level for (A) Craddock100 and (B) Craddock350. Brighter colors represent less differences in the structure of the subnetworks. Gray colors identify smoothing kernels for which no differences were found. we see that: (A) The subnetworks differ slightly for the Craddock100 parcellation and subnetworks remain constant for pairs 6-8 mm, 6-10 mm, 8-10 mm. (B) For the Craddock350 parcellation, subnetwork pais 6-8 mm, 10-12 mm, 16-18 mm, 24-26 mm, and 28-30 mm exihibit the smallest differences.

dataset, and Fig. A20 vs. Fig. A38 for the UCLA dataset). Similarly as with non-adaptive Gaussian smoothing, the least different NBS subnetworks are obtained with consecutive smoothing kernels (Figs. A31 and A39) for both datasets. However, for the ABIDE dataset, the similarity between subnetworks observed at FWHM=0 mm and FWHM=4 mm is higher when the adaptive smoothing is used. This is not a surprise, as the intrinsic smoothness of the raw data is 2-3 mm (Yacoub et al., 2007) and the co-registration step included in our preprocessing pipeline normally further increases the intrinsic smoothness. Therefore, as the smoothness of the data is already close to 4 mm before the spatial smoothing step, practically no additional smoothing is applied at neither FWHM=0 mm nor FWHM=4 mm. The influence of NBS suprathreshold on the subnetwork density is similar indepedent of the smoothing method used (Fig. A28 and Fig. A36).

Likewise, using adaptive smoothing does not change the results of the analysis of thresholded, weighted networks. Due to the large number of significantly different links, it is difficult to observe any differences in the structure of NBS subnetworks observed with adaptive smoothing and non-adaptive Gaussian smoothing (Fig. A2 vs. Fig. A33 for the ABIDE dataset, and Fig. A24 vs. Fig. A41 for the UCLA dataset). Nevertheless, the overall characteristics of the significantly different links (Fig. A3 vs. Fig. A34 for the ABIDE dataset, and Fig. A25 vs. Fig. A42 for the UCLA dataset) and subnetwork similarity (Fig. A4 vs. Fig. A35 for the ABIDE dataset, and Fig. A26 vs. Fig. A43 for the UCLA dataset) remain similar independent of the smoothing method used. For the graph measures, adaptive smoothing yields more consistent results across smoothing levels and thresholds for the degree of the right medial area 8 (Fig. A44) . The effects of network density are not affected by using adaptive smoothing instead of non-adaptive Gaussian smoothing (Fig. A18 vs. Fig. A32 for the ABIDE dataset, and Fig. A23 vs. Fig. A40 for the UCLA dataset)

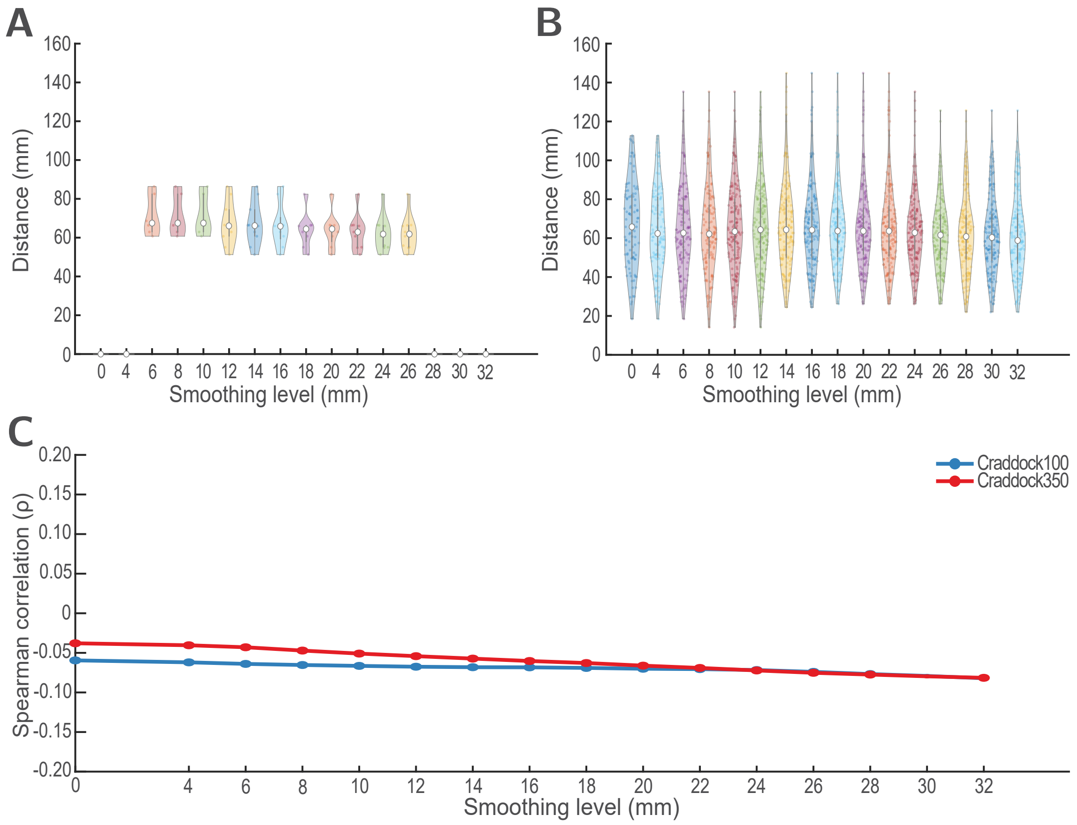

Figure A11: Link-length variation in the between-group differences according to the smoothing kernel (ABIDE dataset) for the Craddock parcellations. Based on the network differences identified by NBS, we see that: (A) The distance profile of the links according to the smoothing level remains almost unaltered for the Craddock100 parcellation. (B) For the Craddock350 parcellation, spatial smoothing alters the distance profile of detected links. We see less number of significantly different long links at small kernels (FWHM$\leq$4 mm) and high kernels (FWHM$>$26mm). (C) Larger kernels are associated with a decrease in the detection of longer links. The slight decline in the value of ρ with increasing FWHM highlights a stronger negative correlation between the length of the links and their F-statistic as the smoothing kernel increases for both parcellations. In other words, using larger kernels decreases the chance of finding long links.

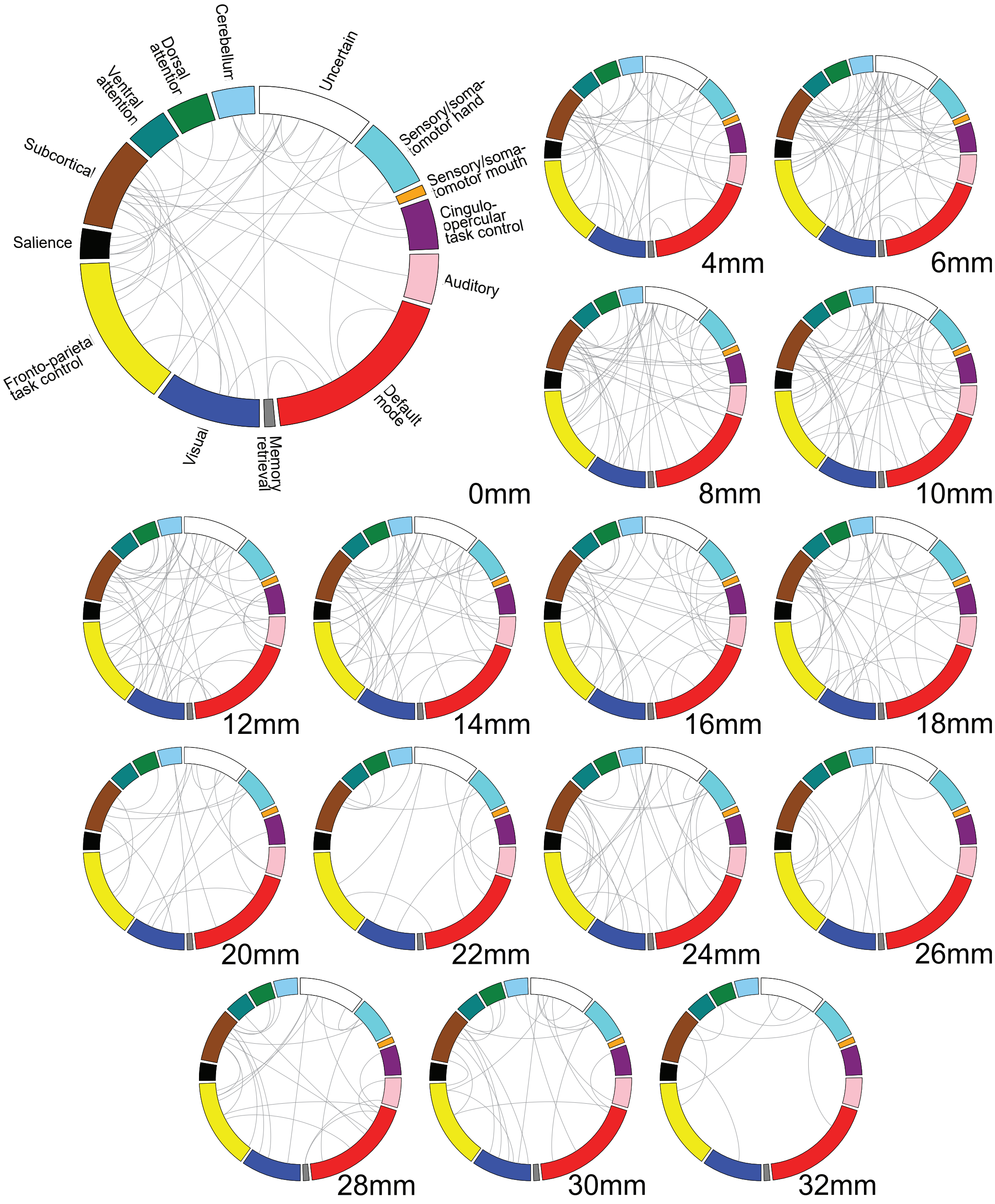

Figure A12: Group-level differences in thresholded, weighted, resting-state functional networks according to the smoothing kernel (ABIDE dataset) for the Craddock100 parcellation. The circos plots show the between-groups connectivity differences identified by permutation tests as described in the section Network comparison of the main article. The nodes are grouped into systems according to Power et al. (2011) and colored accordingly. The choice of smoothing kernel changes the detected connectivity differences. The connections found at low kernels differ drastically from those at high kernels in terms of comprised links. Subnetwork density decreases as the smoothing kernel increases.

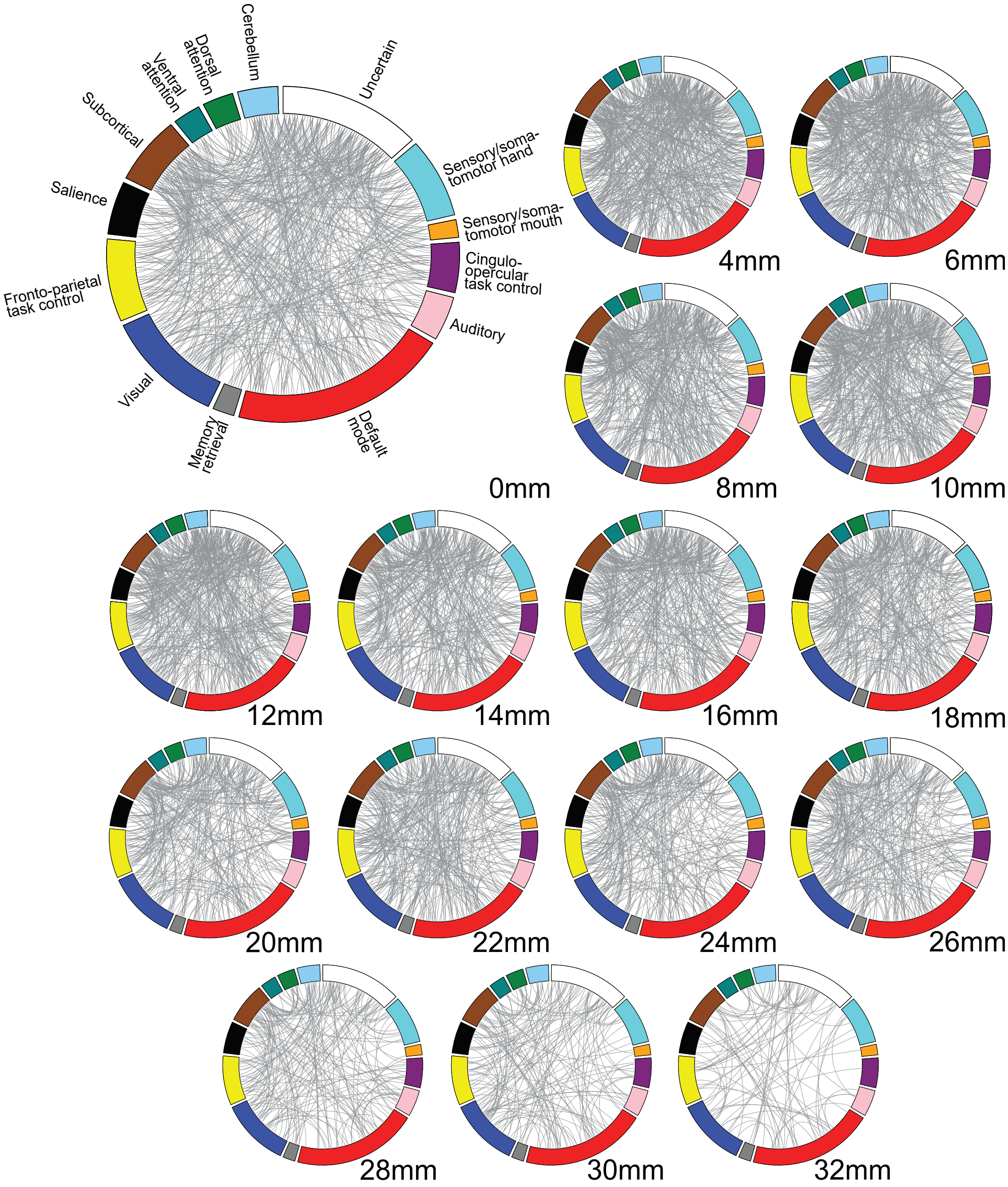

Figure A13: Group-level differences in thresholded, weighted, resting-state functional networks according to the smoothing kernel (ABIDE dataset) for the Craddock350 parcellation. The circos plots show the between-groups connectivity differences identified by permutation tests as described in the section Network comparison of the main article. The nodes are grouped into systems according to Power et al. (2011) and colored accordingly. The choice of smoothing kernel changes the detected connectivity differences. The large number of significantly different links makes difficult to detect possible patterns. Nevertheless we notice fewer links are detected at high kernels (e.g. 30 and 32 mm).

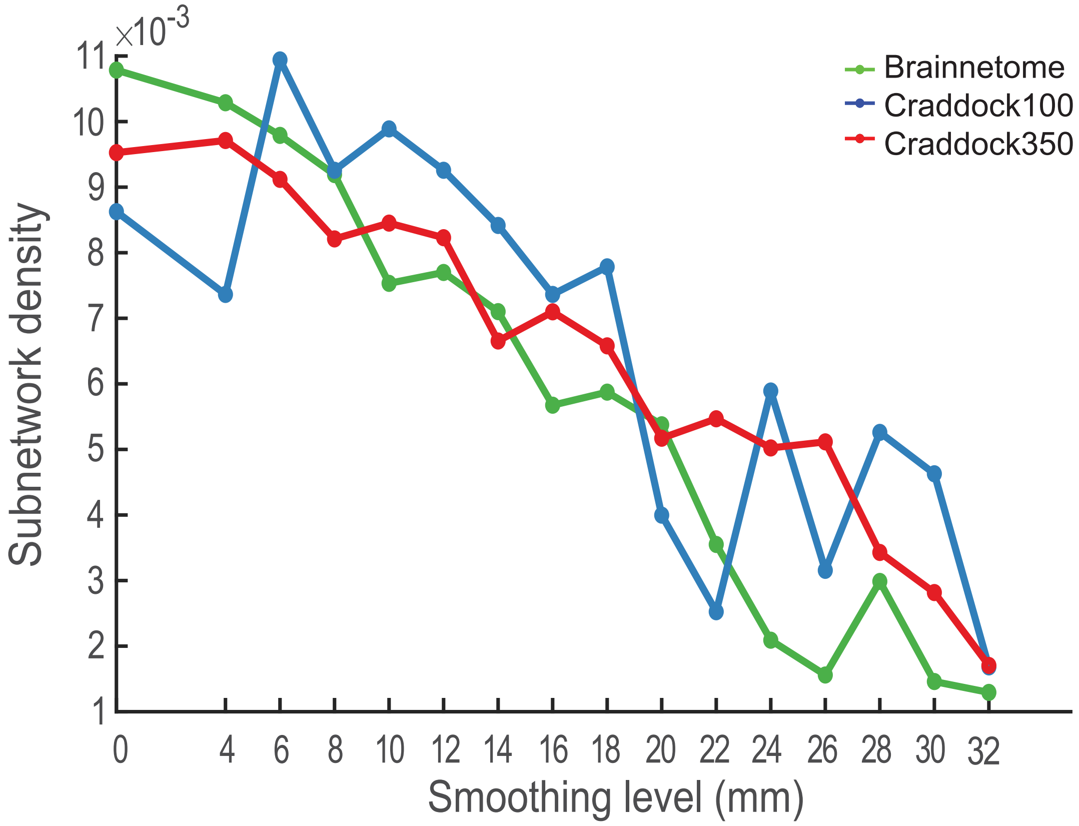

Figure A14: Variation of between-group differences according to the smoothing kernel for different parcellations (ABIDE dataset). Based on the network differences identified by the permutation tests, we see that the choice of smoothing kernel affects the number of links. In general, the number of significantly different links decreases with the increase in the smoothing kernel width. This trend is evident for the Brainnetome and Craddock350 parcellations. For the Craddock100 parcellation, the trend is more variable, with sudden increases in the number of detected links (e.g. 6 mm and 24 mm).

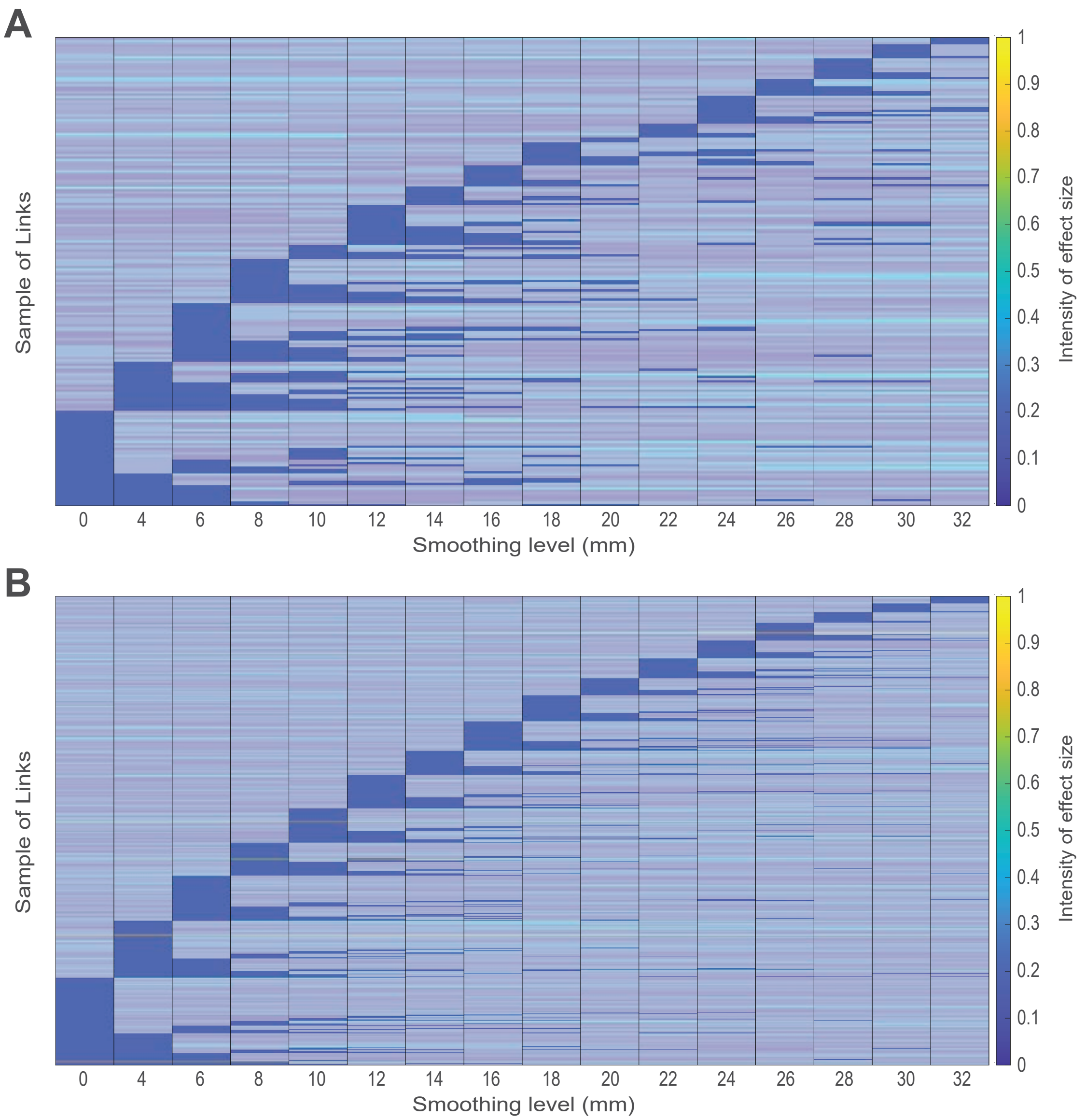

Figure A15: Effect-size variation in the between-group differences according to the smoothing kernel (ABIDE dataset) for the Craddock parcellations. Based on the network differences identified by the permutation tests, we see that the effect sizes of the significantly different links change depending on the smoothing kernel for both parcellations, (A) Craddock100 and (B) Craddock350. The figure shows the smoothing kernels on the x-axis, ordered from smallest (0 mm) to largest (32 mm). In the y-axis, the figure shows the links which the permutation tests identified as different between groups at some kernel. The links (y-axis) are organized according to the smallest kernel in which they are detected and the number of smoothing levels in which they appear statistically significant (corrected p-value $\alpha<0.05$); for example, all links which are significant at 0 mm are shown at the bottom of the plot, then on top of them, we show all links which are significant at 4mm, but are not significant at 0mm. This organization follows until all smoothing levels are shown. The plot highlights those kernels at which the links are found significant. No links are detected at all smoothing levels and few links have a strong effect size. Some links are detected at kernels that are not consecutive, highlighting the unsystematic effect of spatial smoothing.

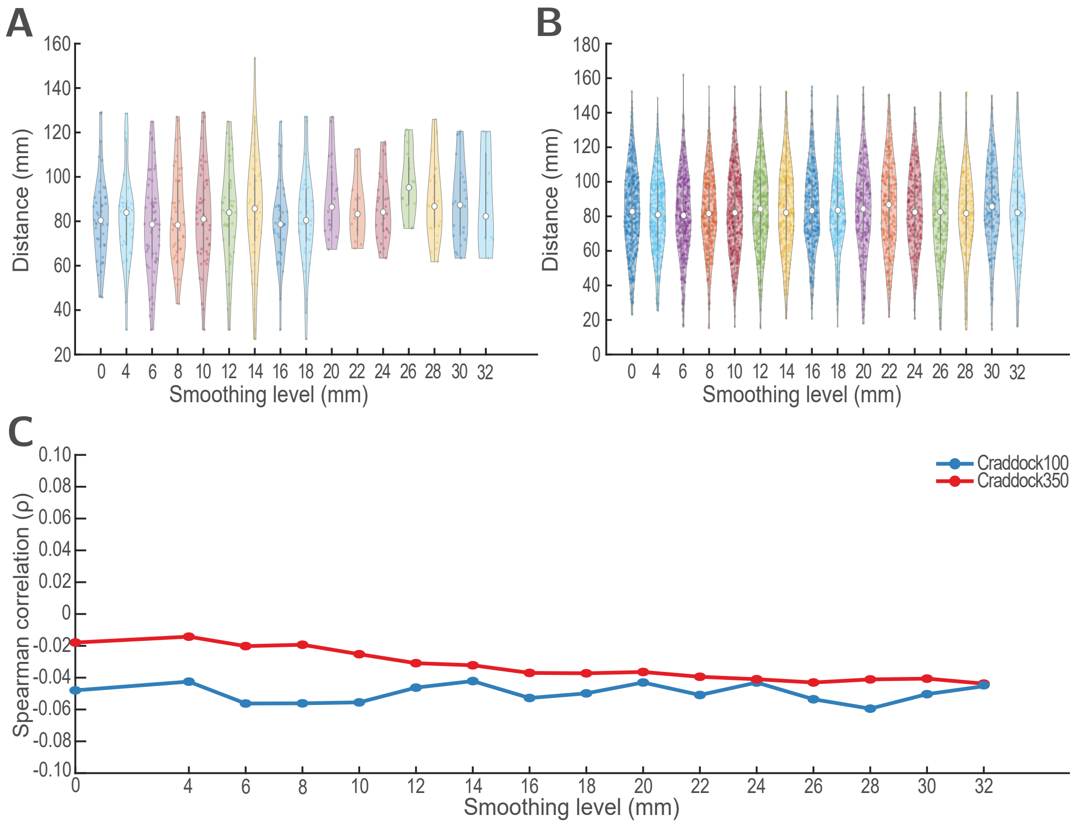

Figure A16: Link-length variation in the between-group differences according to the smoothing kernel (ABIDE dataset) for the Craddock parcellations. Based on the network differences identified by the permutation tests, we see that: (A) For the Craddock100 parcellation, we see less number of significantly different short links at high kernels (FWHM $\geq$ 20 mm). (B) For the Craddock350 parcellation, spatial smoothing alters the distance profile of detected links with no clear pattern (C) Larger kernels are associated with a decrease in the detection of longer links. The slight decline in the value of ρ with increasing FWHM highlights a stronger negative correlation between the length of the links and their T-statistic as the smoothing kernel increases for the Craddock350 parcellation. On the other hand, for the Craddock100 parcellation, the trend seems to vary between decrease (e.g from 0 to 8 mm) and increase (e.g. from 8 to 14 mm).

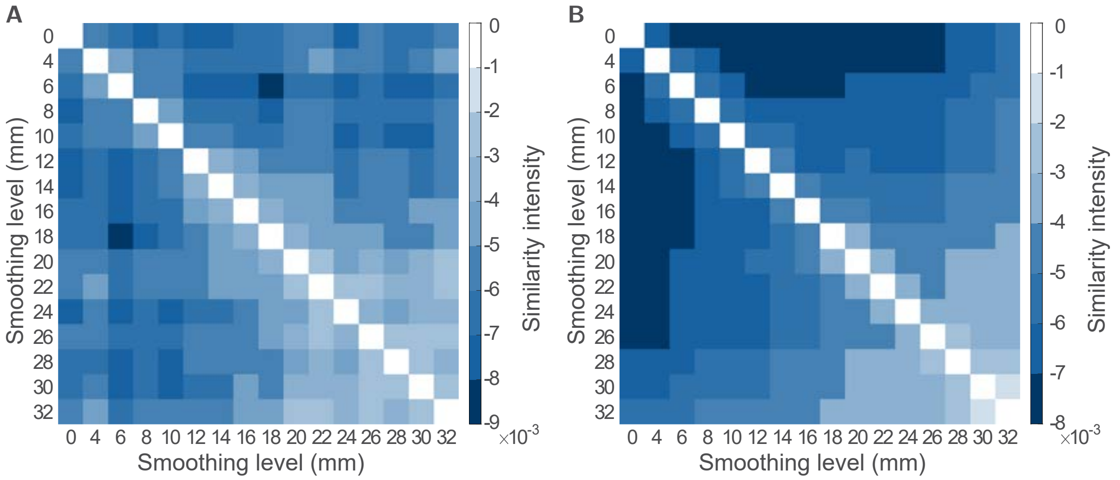

Figure A17: Similarity matrix between the identified group-level network differences at each smoothing level for for thresholded, weighted matrices. Brighter colors represent less differences in the structure of the significantly different links. We see that: (A) For Craddock100, we see the smallest differences between large kernels (FWHM $\geq$ 20 mm). (B) This pattern is more evident for Craddock350 parcellation. Conversely, the most dissimilar networks are found in the small kernels (i.e. 0, 4, and 6 mm).

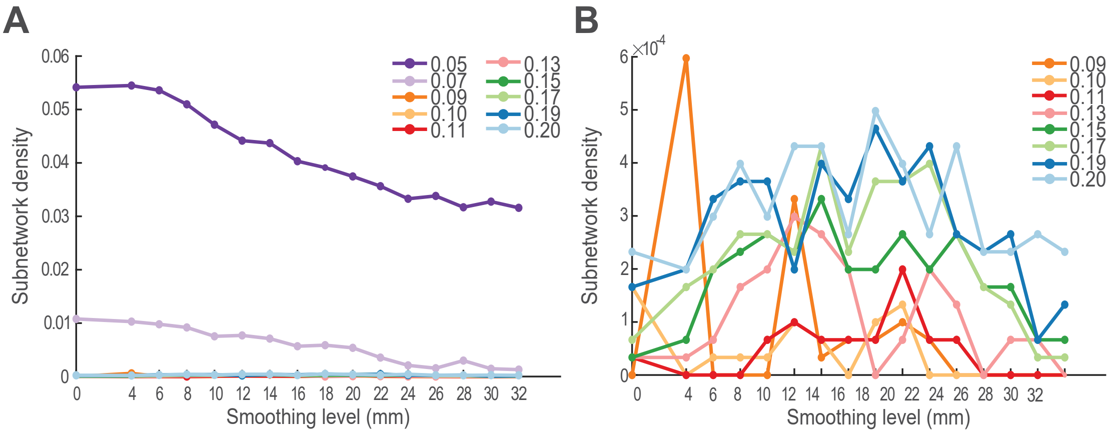

Figure A18: Network density thresholds influence the number of significantly different links comprising the set of between-group differences. We compare the number of links yielded by the permutation tests at 10 different threshold values (ABIDE dataset, Brainnetome parcellation). We see that: (A) for sparse networks, the number of significant links decreases as the smoothing level increases. (B) the number of significantly different links found at each smoothing kernel varies unsystematically depending on the network density threshold.

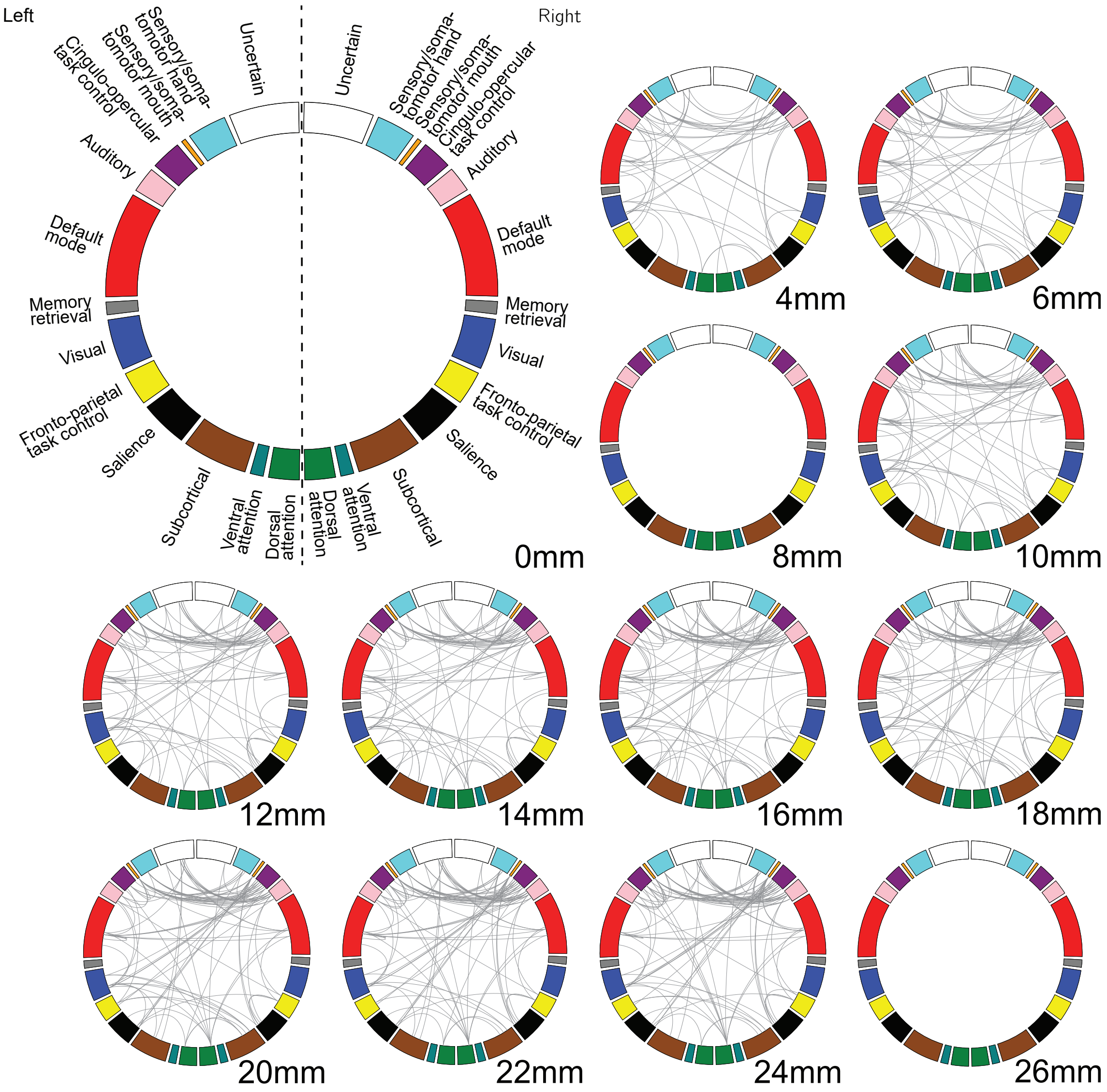

Figure A19: Group-level differences in resting-state functional networks for different smoothing kernels (UCLA dataset, Brainnetome parcellation). The circos plots show the between-groups connectivity differences identified by NBS as described in the Network comparison section of the main article. The nodes are grouped into systems according to Power et al. (2011), colored accordingly, and split in left and right hemispheres. The choice of smoothing kernel changes the detected connectivity differences. Between-group differences are not detected for kernels larger than FWHM=26 mm. No links are detected at FWHM=0 mm and FWHM=8 mm.

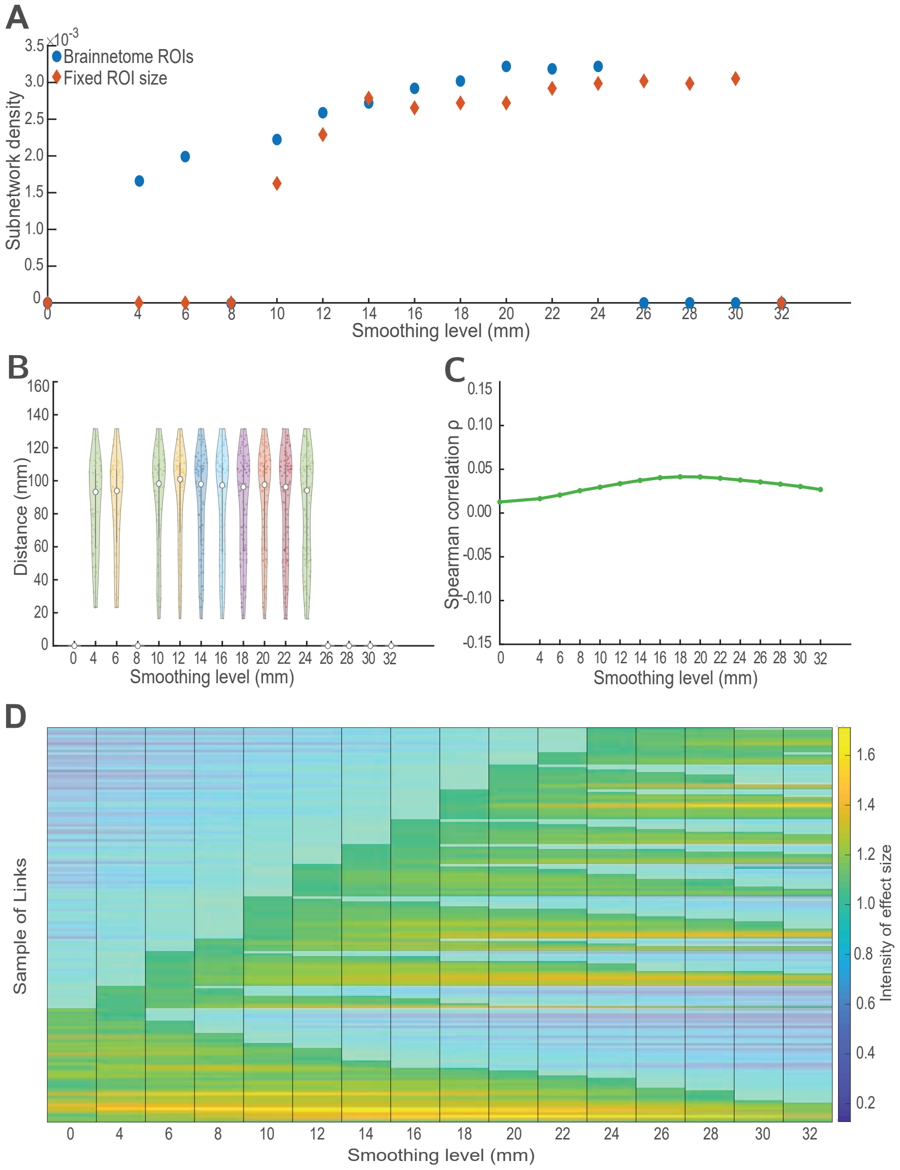

Figure A20: Characteristics of between-group differences according to the smoothing kernel for the UCLA dataset, Brainnetome parcellation. Based on the subnetworks identified by NBS, we see that: (A) The choice of smoothing kernel affects the number of links of the subnetworks and its effects are independent of ROI size. Initially, the number of links in the subnetwork increases to then decrease abruptly (FWHM=24 mm). This pattern is present even when the ROIs are artificially constructed as spheres of constant ratios. (B) Spatial smoothing alters the distance profile of detected links. (C) Larger kernels are associated with both, an increase (from 0 to 18 mm), and a decrease (from 20 to 32 mm) in the detection of longer links. A decline in the value of ρ with increasing FWHM highlights a stronger negative correlation between the length of the links and their F-statistic, in other words, using larger kernels will decrease the chances of finding long links. Conversely, an increase in the value of ρ with increasing FWHM highlights a stronger positive correlation between the length of the links and their F-statistic, in other words, using larger kernels will increase the chances of finding long links. (D) Effect sizes of the subnetwork links change depending on the smoothing kernel. The figure shows shows the smoothing kernels on the x-axis, ordered from smallest (0 mm) to largest (32 mm). In the y-axis, the figure shows the links which NBS identified as different between groups at some kernel. The links (y-axis) are organized according to the smallest kernel in which they are detected and the number of smoothing levels in which they appear statistically significant (α<0.05); for example, all links which are significant at 0 mm are shown at the bottom of the plot, then on top of them, we show all links which are significant at 4mm, but are not significant at 0mm. This organization follows until all smoothing levels are shown. The plot highlights those kernels at which the links are found significant. No links are detected at all smoothing levels. Conversely, some links are only observed when a particular kernel is used.

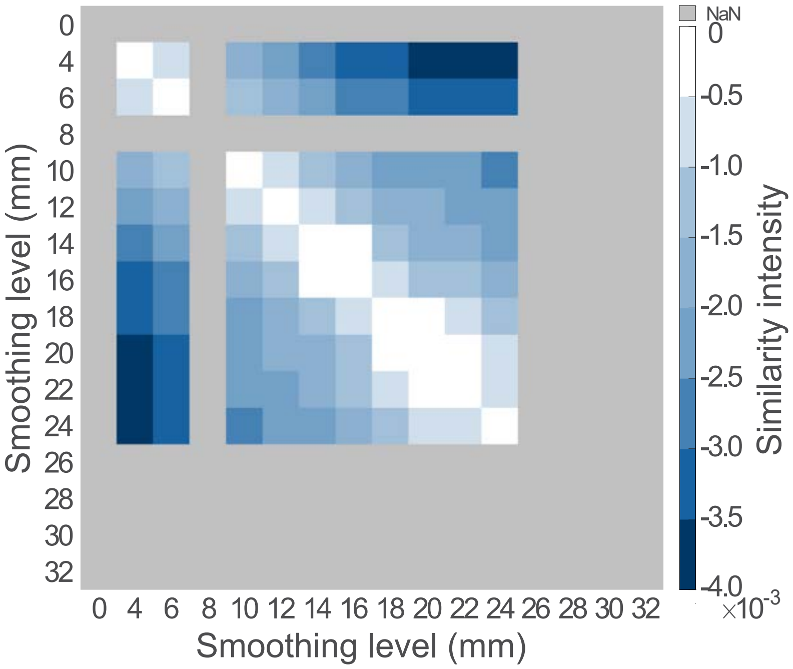

Figure A21: Similarity matrix between the identified subnetwork differences for each smoothing level (UCLA dataset). Brighter colors represent less differences in the structure of the subnetworks. Gray colors show smoothing kernels for which no differences are found. Subnetworks pairs 14-16 mm, 18-20 mm, and 20-22 mm show the smallest differences between them.

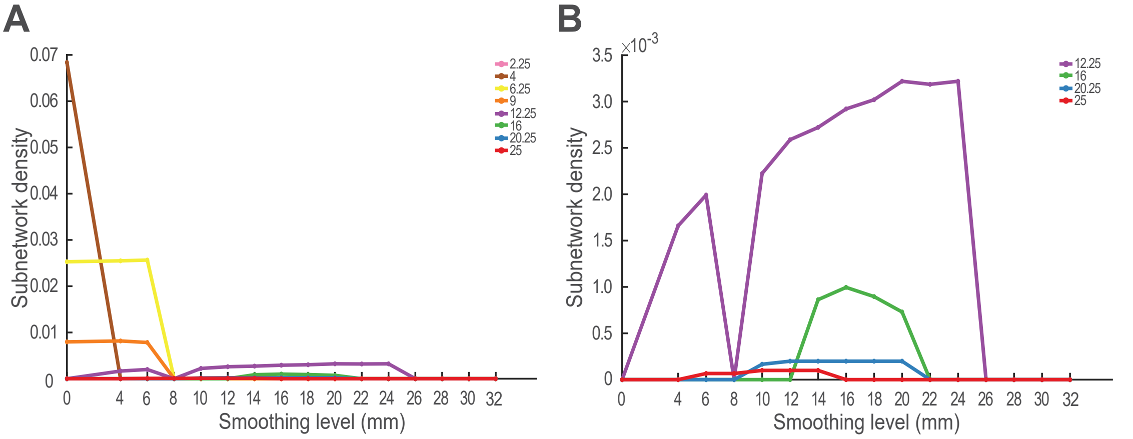

Figure A22: NBS suprathresholds influence the number of significantly different links comprising the subnetwork. (A) We compare the number of links yielded by NBS at 8 different suprathreshold values (UCLA dataset). Surprisingly, smaller suprathresholds yield subnetworks for only few kernels in which the density does not vary. (B) Larger suprathresholds have similar effects to the reported in the Results section of the main article, reporting an increase in the subnetwork density when the smoothing kernel width is larger. However, this trend is inverted at some inflexion point, which varies for each suprathreshold. The trend's U-inverted shape of the spatial smoothing effects can be seen for 12.25, 16, 20.25, and 25 suprathresholds.

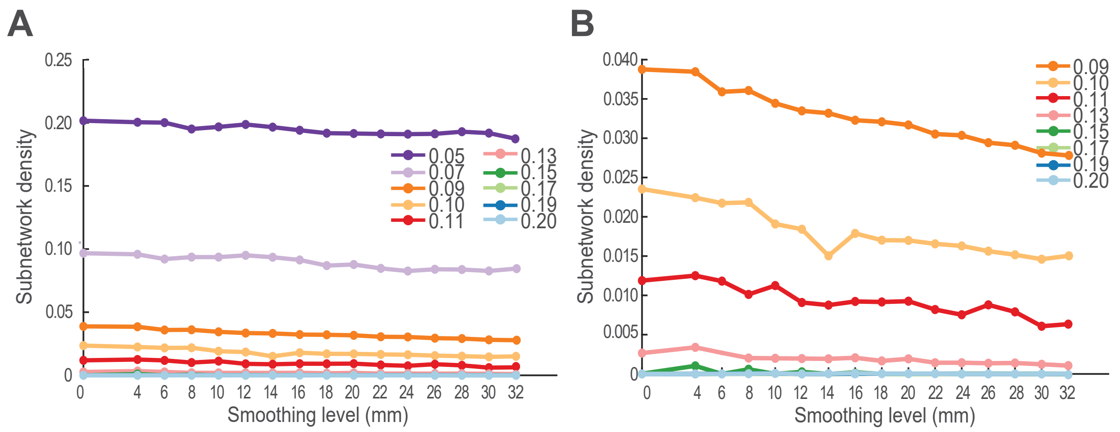

Figure A23: Network density thresholds influence the number of significantly different links comprising the set of between-group differences. We compare the number of links yielded by the permutation tests at 10 different threshold values (UCLA dataset). In general, the number of significantly different links decreases with the increment in the smoothing kernel width for all tested thresholds.

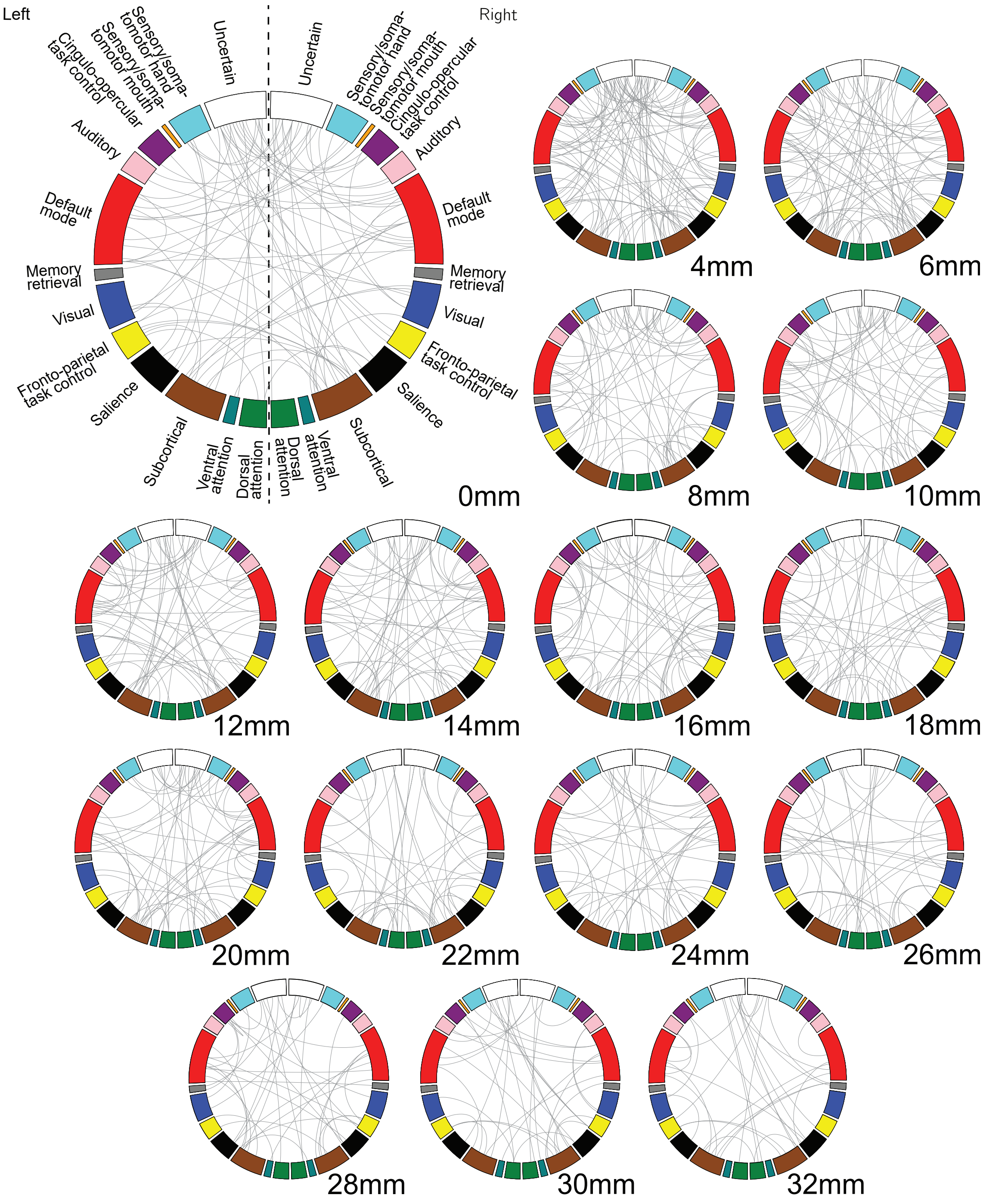

Figure A24: Group-level differences in thresholded, weighted resting-state functional networks for different smoothing kernels (UCLA dataset, Brainnetome parcellation). The circos plots show the between-groups connectivity differences identified by permutation tests as described in the Network comparison section of the main article. The nodes are grouped into systems according to Power et al. (2011), colored accordingly, and split in left and right hemispheres. The choice of smoothing kernel changes the detected connectivity differences.

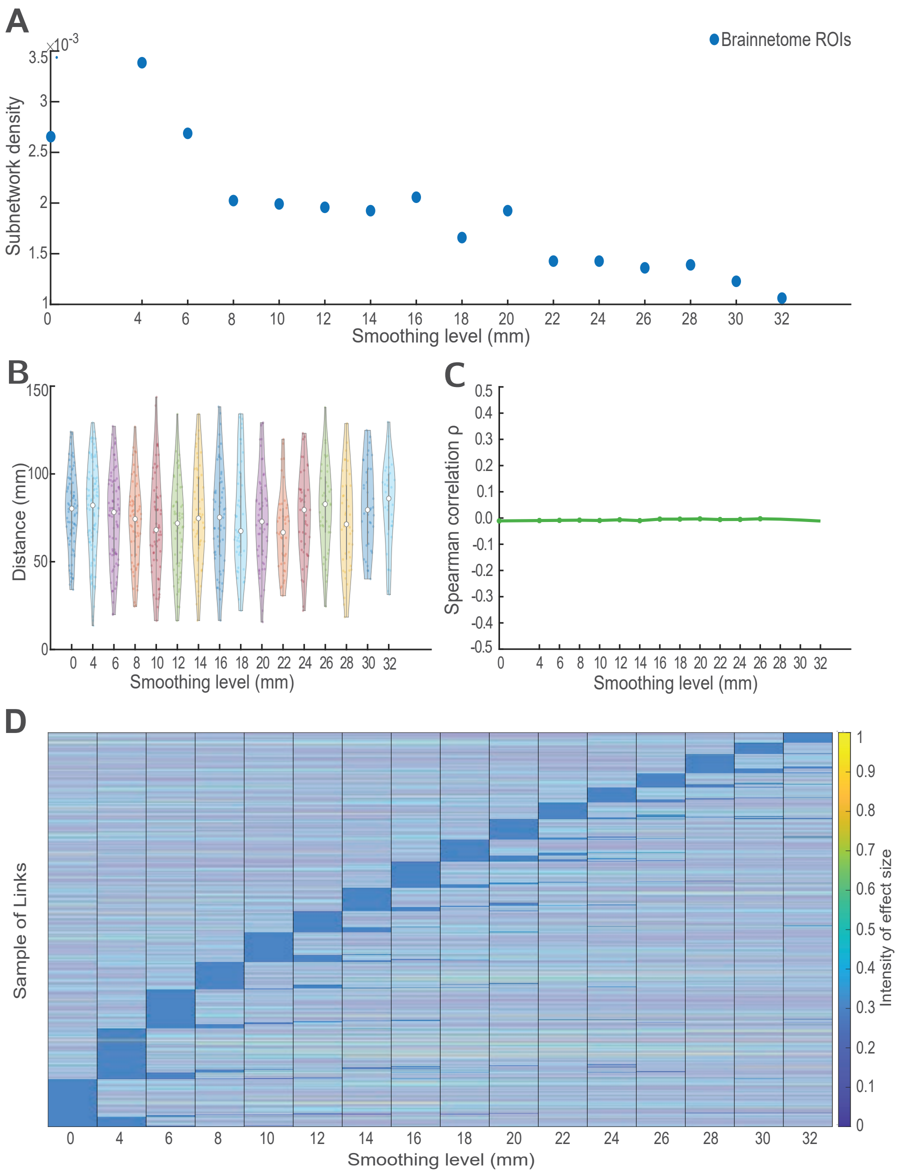

Figure A25: Characteristics of between-group differences according to the smoothing kernel for thresholded, weighted networks (UCLA dataset, Brainnetome parcellation). Based on the network differences identified by the permutation tests, we see that: (A) The choice of smoothing kernel affects the number of significantly different links. In general, the number of significantly different links decreases. (B) Spatial smoothing alters the distance profile of detected links with no clear pattern. (C) No variation is shown between the value of ρ and FWHM, i.e. no relationship is found between the smoothing kernel and the longitude of the significant links found. (D) Effect sizes of the significantly different links change depending on the smoothing kernel. The figure shows the smoothing kernels on the x-axis, ordered from smallest (0 mm) to largest (32 mm). In the y-axis, the figure shows the links which permutation tests yielded to be different between groups at some kernel. The links (y-axis) are organized according to the smallest kernel in which they are detected and the number of smoothing levels in which they appear statistically significant (corrected p-value α<0.05); for example, all links which are significant at 0 mm are shown at the bottom of the plot, then on top of them, we show all links which are significant at 4mm, but are not significant at 0mm. This organization follows until all smoothing levels are shown. The plot highlights those kernels at which the links are found significant. No links are detected at all smoothing levels. Some links are detected at kernels that are not consecutive or at single kernels.

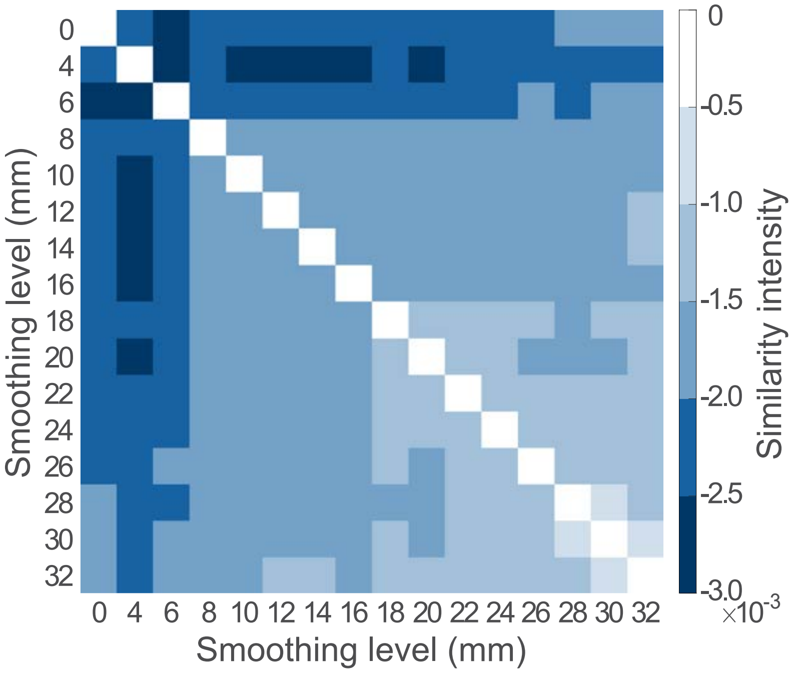

Figure A26: Similarity matrix between the identified subnetwork differences at each smoothing level for thresholded, weighted networks of the UCLA dataset. Brighter colors represent less differences in the structure of the subnetworks. Subnetworks pairs 0-6 mm, 4-6 mm, 4-10 mm, 4-12 mm, 4-14 mm, 4-16 mm, and 4-20 mm show the largest differences.

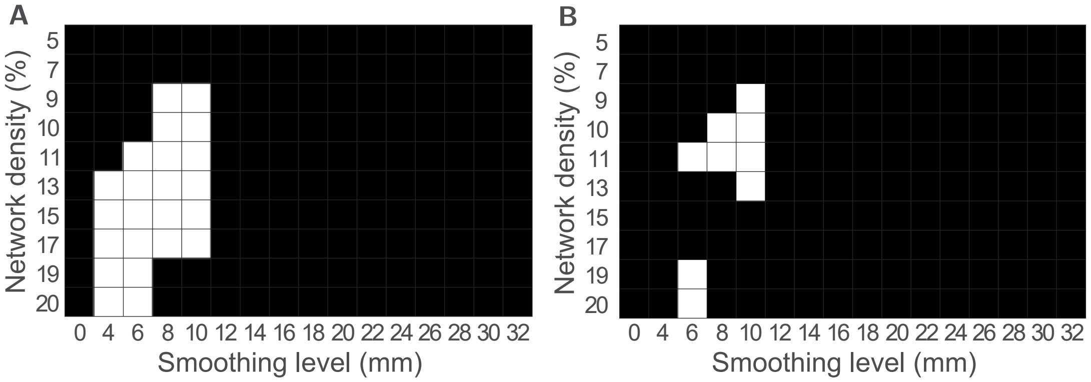

Figure A27: Significant differences are found in the degree of nodes (A) right medial area 8 and (B) left caudal temporal thalamus in the UCLA dataset. The differences are detected consistently across different network densities and for commonly used kernels.

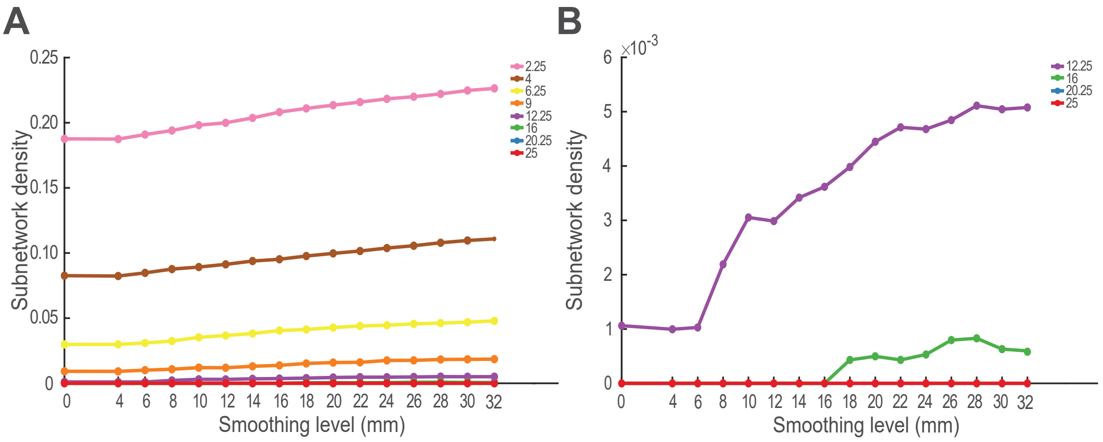

Figure A28: NBS suprathresholds influence the number of significantly different links comprising the subnetwork (ABIDE dataset, adaptive smoothing). (A) We compare the number of links yielded by NBS at 8 different suprathreshold values. Similar to the results of non-adaptive smoothing, smaller suprathresholds yield denser networks as more links are selected, making the connected component larger. For smaller suprathresholds, larger smoothing kernels yield denser subnetworks. (B) Only some larger suprathresholds detect some links in the subnetwork.

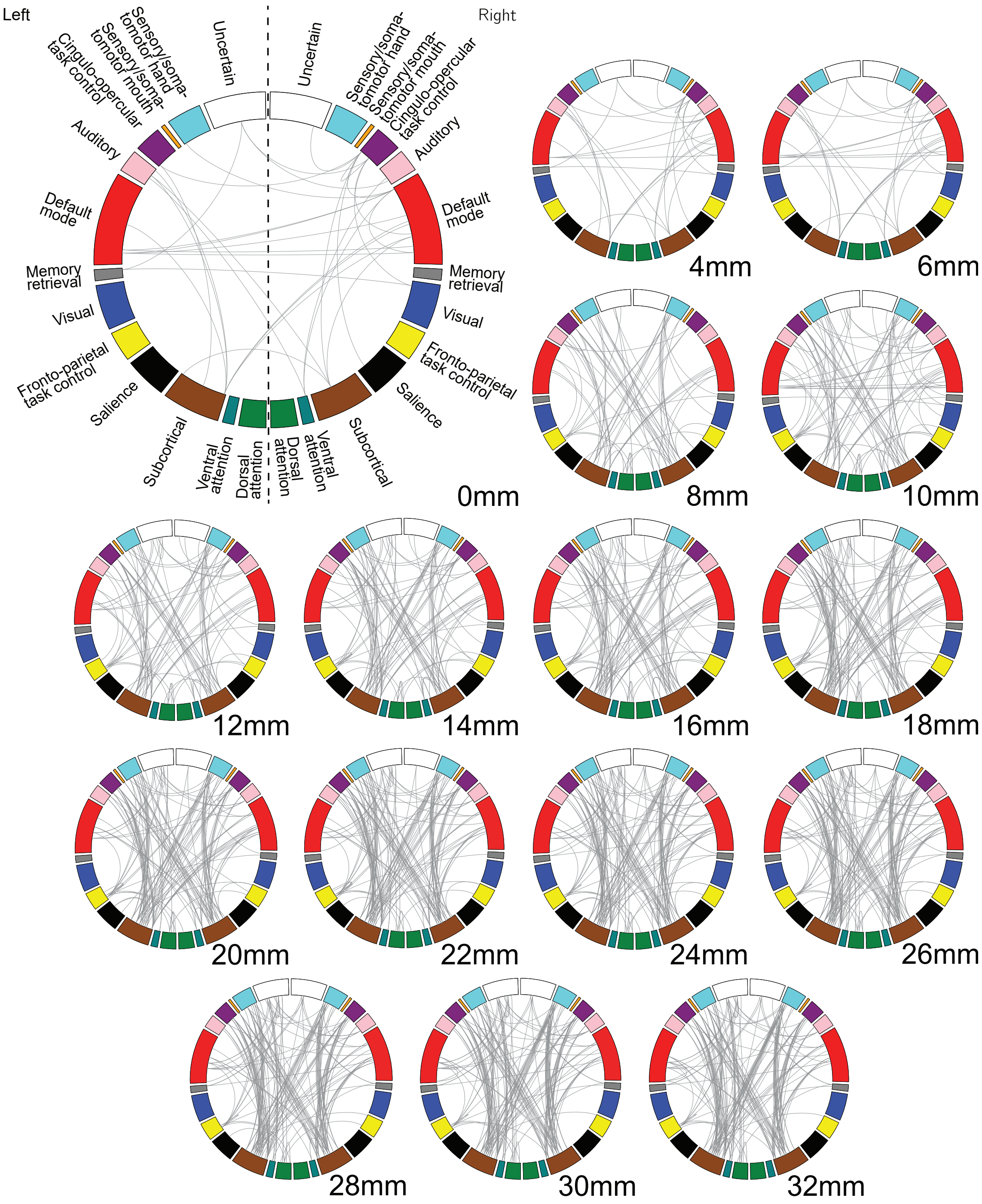

Figure A29: Group-level differences in resting-state functional networks for different smoothing kernels (ABIDE dataset, adaptive smoothing). The circos plots show the between-groups connectivity differences identified by NBS. The nodes are grouped into systems following Power et al. (2011), colored accordingly, and split into the left and right hemispheres. The width of the smoothing kernel changes the detected connectivity differences. The connections found at low kernel widths differ from those at high kernel widths in terms of structure. Subnetworks detected with this method are similar to the non-adaptive smoothing. However, subnetworks for kernels FWHM=4 mm and FWHM=6 mm differ structurally.

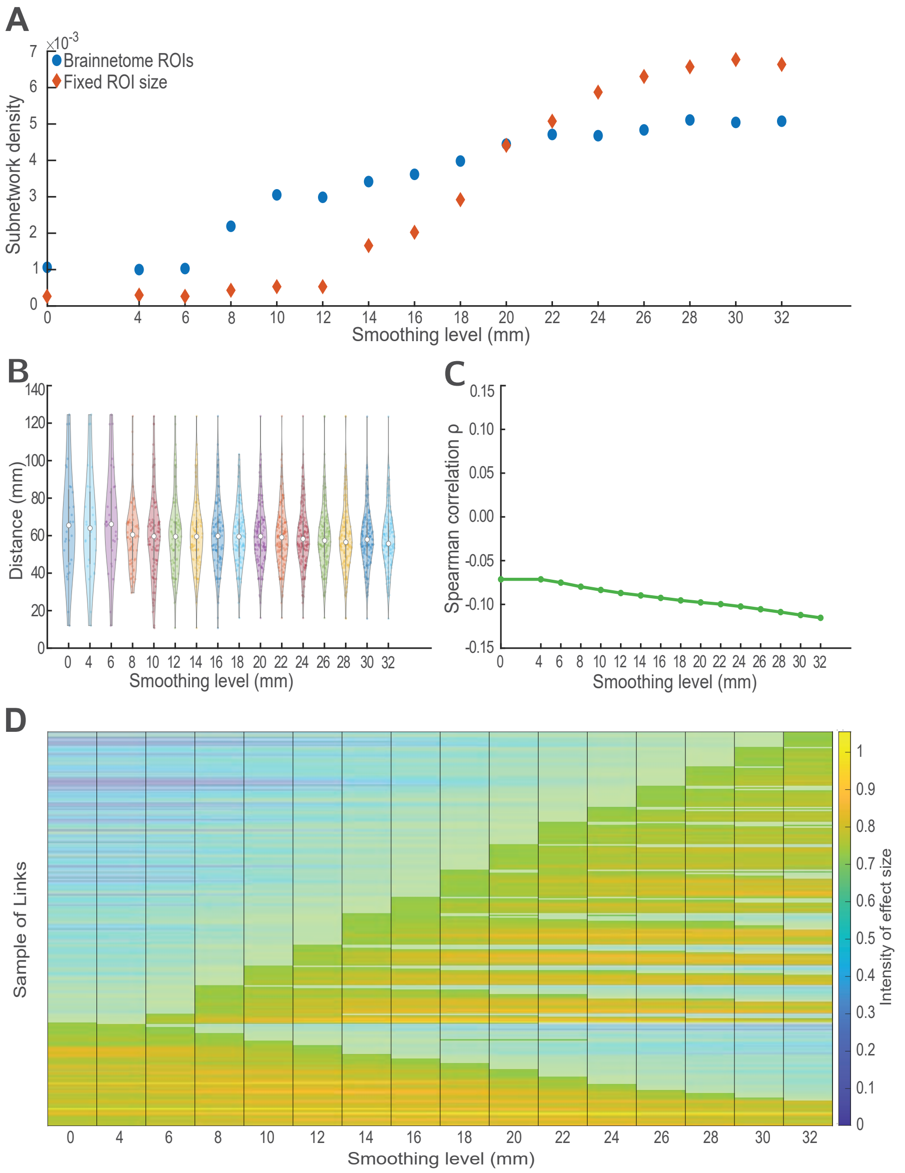

Figure A30: Characteristics of between-group differences for each smoothing kernel (ABIDE dataset, adaptive smoothing). Based on the subnetworks identified by NBS, we see that: (A) The choice of smoothing kernel affects the number of links of the subnetworks and its effects are independent of ROI size. The number of links in the subnetwork increases as the smoothing level is incremented. This pattern is present even when the ROIs are artificially constructed as spheres of constant ratios. (B) Spatial smoothing alters the distance profile of detected links. (C) Larger kernels are associated with a decrease in the detection of longer links. A decline in the value of ρ with increasing FWHM highlights a stronger negative correlation between the length of the links and their F-statistic. In other words, using larger kernels decreases the chance of finding long links. (D) Effect sizes of the subnetwork links change depending on the smoothing kernel. The y-axis shows the links which NBS identified as different between groups at some kernel. The links (y-axis) are organized according to the smallest kernel in which they are detected and the number of smoothing levels in which they appear statistically significant (α<0.05); for example, all links which are significant at 0 mm are shown at the bottom of the plot, then on top of them, we show all links which are significant at 4mm, but are not significant at 0mm. This organization follows until all smoothing levels are shown. The plot highlights those kernels at which the links are found significant. 17 links are detected at all smoothing levels. Conversely, some links are only observed when a particular kernel is used despite having a large effect at other kernels.

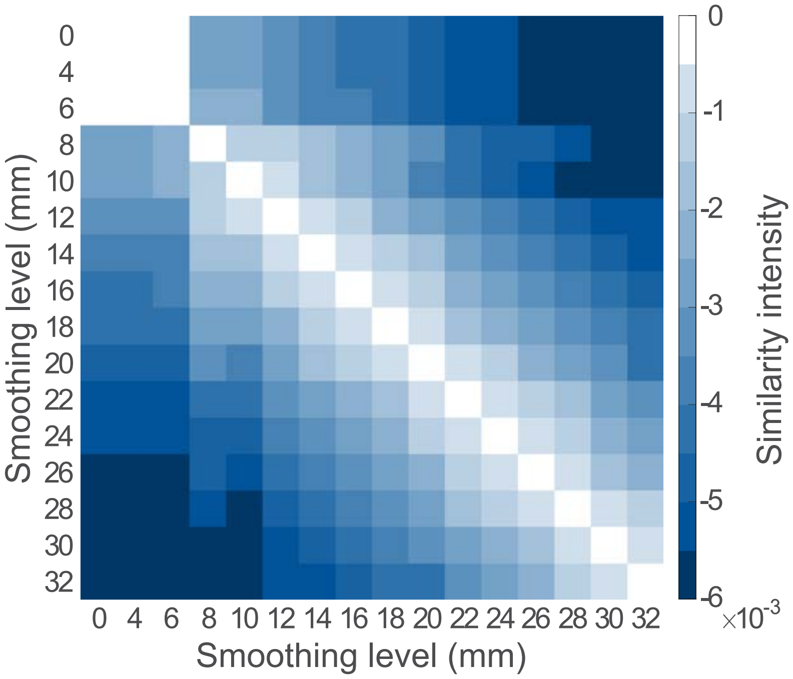

Figure A31: Similarity matrix between the identified subnetwork differences at each smoothing level (ABIDE dataset, adaptive smoothing). Brighter colors represent less differences in the structure of the subnetworks. Subnetworks pairs 0-4 mm and 0-6 mm are more similar than the rest of kernels.

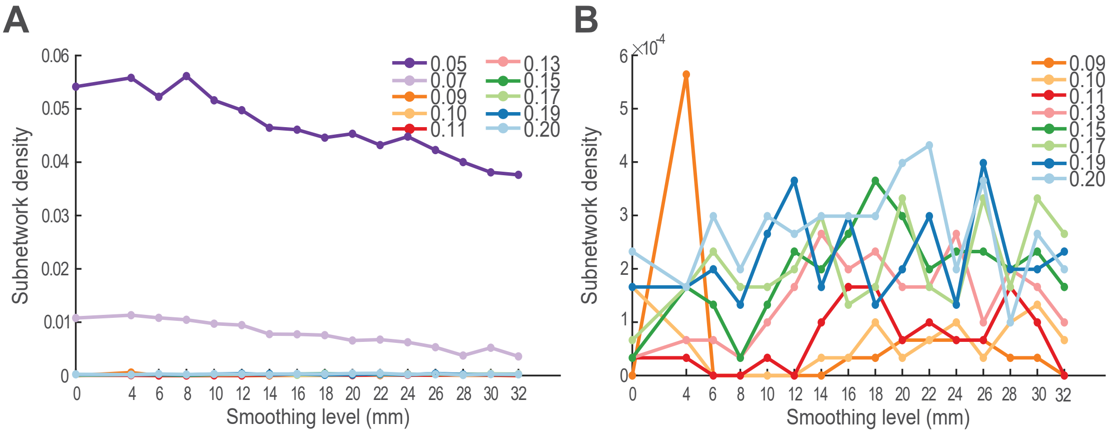

Figure A32: Network density thresholds influence the number of significantly different links comprising the set of between-group differences (ABIDE dataset, adaptative smoothing). We compare the number of links yielded by the permutation tests at 10 different threshold values (ABIDE dataset, Brainnetome parcellation, adaptive smoothing). Similar to the results obtained with non-adaptive strategies, we see that the number of significantly different links found at each smoothing kernel varies unsystematically depending on the network density threshold.

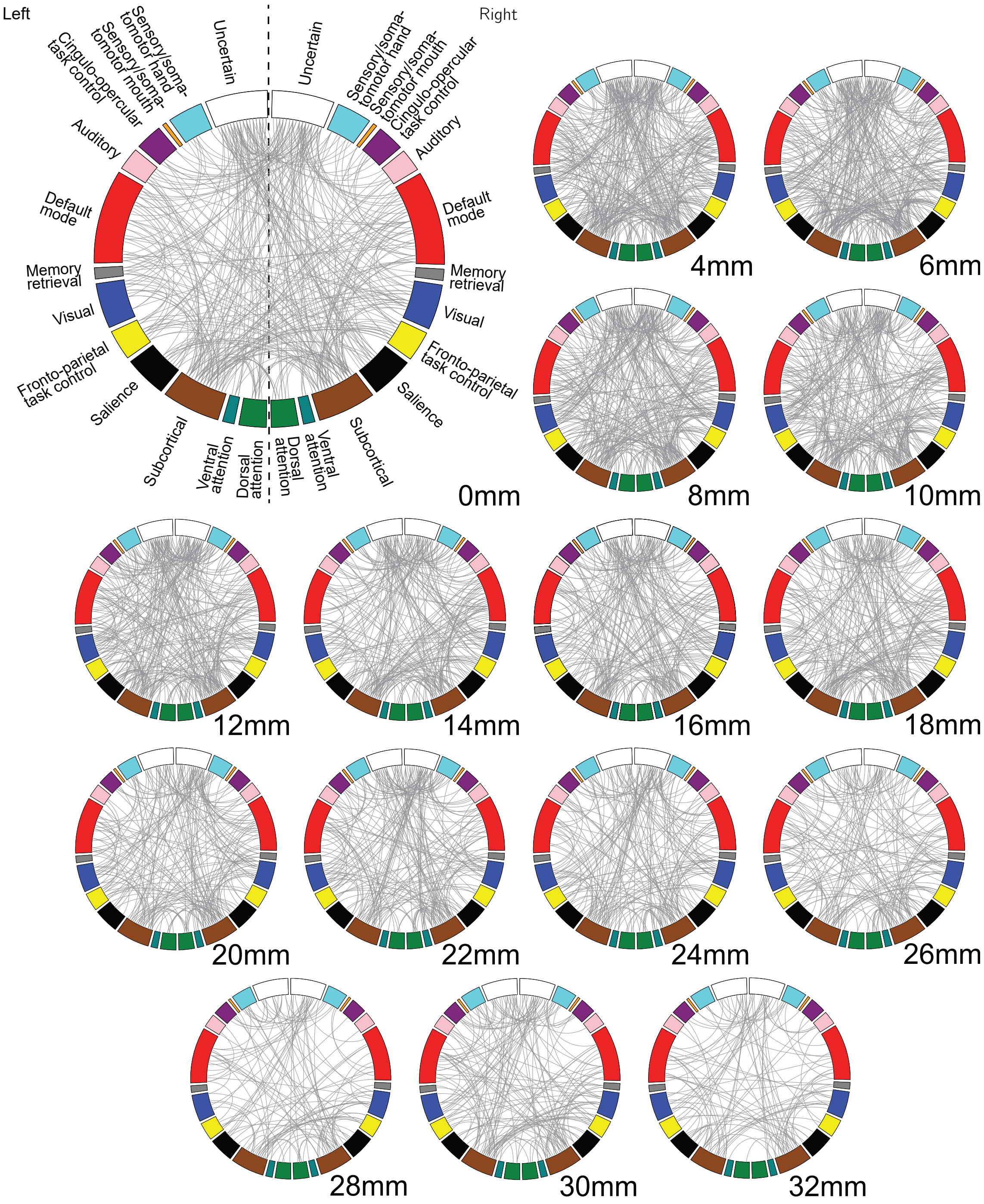

Figure A33: Group-level differences in resting-state functional networks for different smoothing kernels, adaptive smoothing. The circos plots show the between-groups connectivity differences identified by permutations. The nodes are grouped into systems following Power et al. (2011), colored accordingly, and split into the left and right hemispheres. The width of the smoothing kernel changes the detected connectivity differences. The connections found at low kernel widths differ from those at high kernel widths in terms of structure.

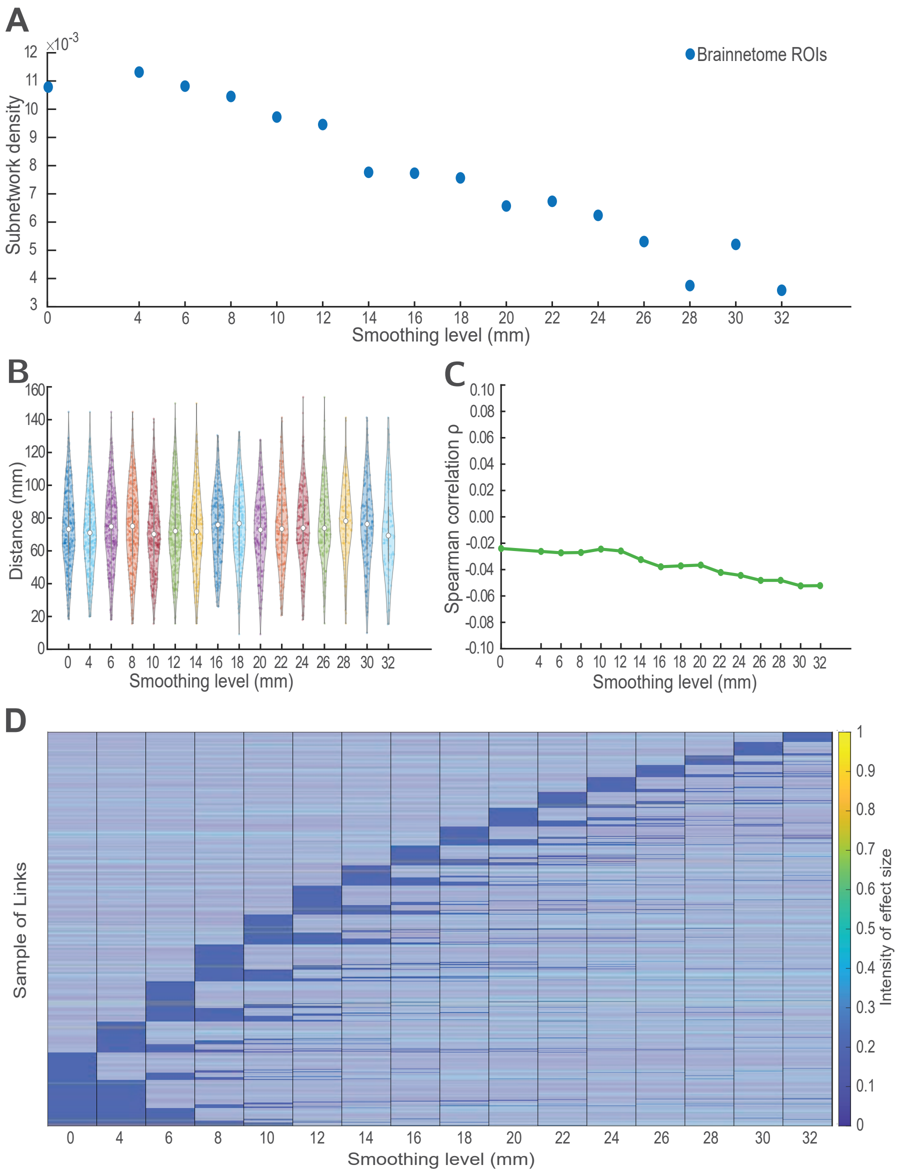

Figure A34: Characteristics of between-group differences according to the smoothing kernel, (ABIDE dataset, adaptive smoothing). Based on the subnetworks identified by permutations, we see that: (A) The choice of smoothing kernel affects the number of links of the subnetworks and its effects are independent of ROI size. The number of links in the subnetwork decreases as the smoothing kernel increases. (B) Spatial smoothing alters the distance profile of detected links at commonly used kernels with no discernible pattern. (C) Larger kernels are associated with a decrease in the detection of longer links. A decline in the value of ρ with increasing FWHM highlights a stronger negative correlation between the length of the links and their T-statistic, in other words, using larger kernels decreases the chance of finding long links. (D) Effect sizes of the subnetwork links change depending on the smoothing kernel. The figure shows shows the smoothing kernels on the x-axis, ordered from smallest (0 mm) to largest (32 mm). In the y-axis, the figure shows the links which the permutation method identified differences between groups at some kernel. The links (y-axis) are organized according to the smallest kernel in which they are detected and the number of smoothing levels in which they appear statistically significant (α<0.05); for example, all links which are significant at 0 mm are shown at the bottom of the plot, then on top of them, we show all links which are significant at 4mm, but are not significant at 0mm. This organization follows until all smoothing levels are shown. The plot highlights those kernels at which the links are found significant. No links are detected at all smoothing levels. Conversely, some links are only observed when a particular kernel is used. Moreover, some links are detected at kernels that are not consecutive, evidencing the unpredictability of the effects. In general, the effect sizes are small.

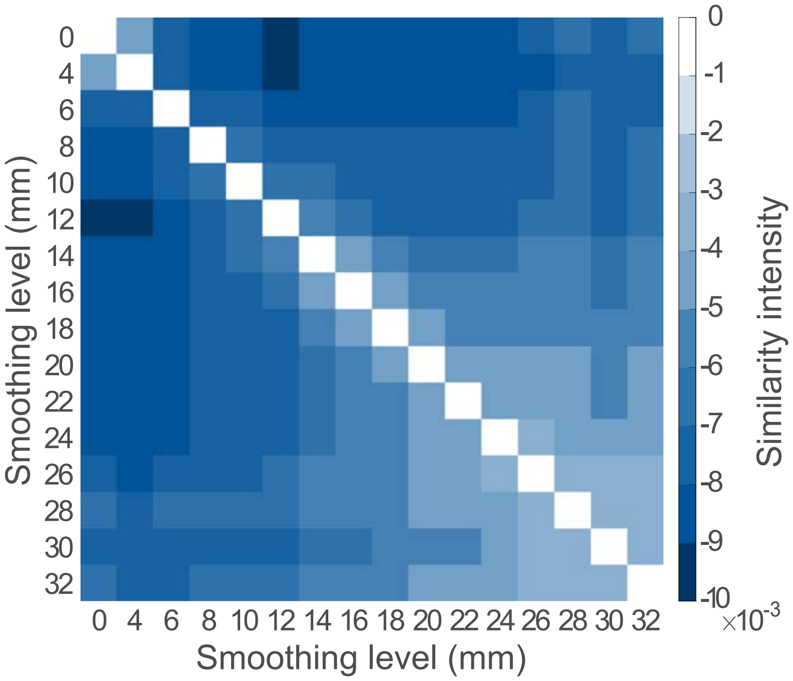

Figure A35: Similarity matrix between the identified group-level network differences at each smoothing level for thresholded, weighted matrices (ABIDE dataset, adaptive smoothing). Brighter colors represent less differences in the structure of the links. Surprisingly, subnetwork pairs 0-12 mm and 4-12 mm exhibit the largest difference, even larger than e.g. subnetwork pair 0-30 mm. This evidences the variation in the detected links.

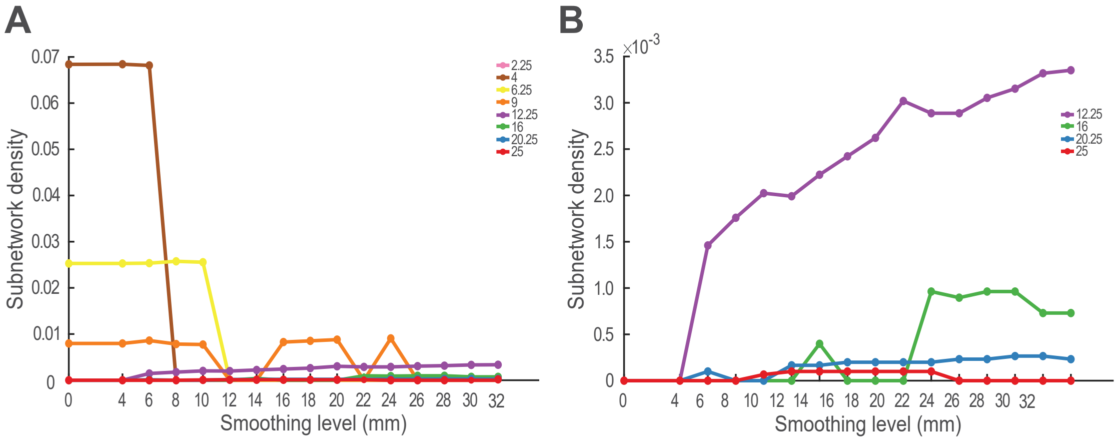

Figure A36: NBS suprathresholds influence the number of significantly different links comprising the subnetwork. (A) We compare the number of links yielded by NBS at 8 different suprathreshold values (UCLA dataset, adaptive smoothing). Surprisingly, smaller suprathresholds yield subnetworks for only few kernels. (B) Larger suprathresholds have similar effects, although high suprathresholds also present a decline in this trend (e.g. suprathreshold 25).

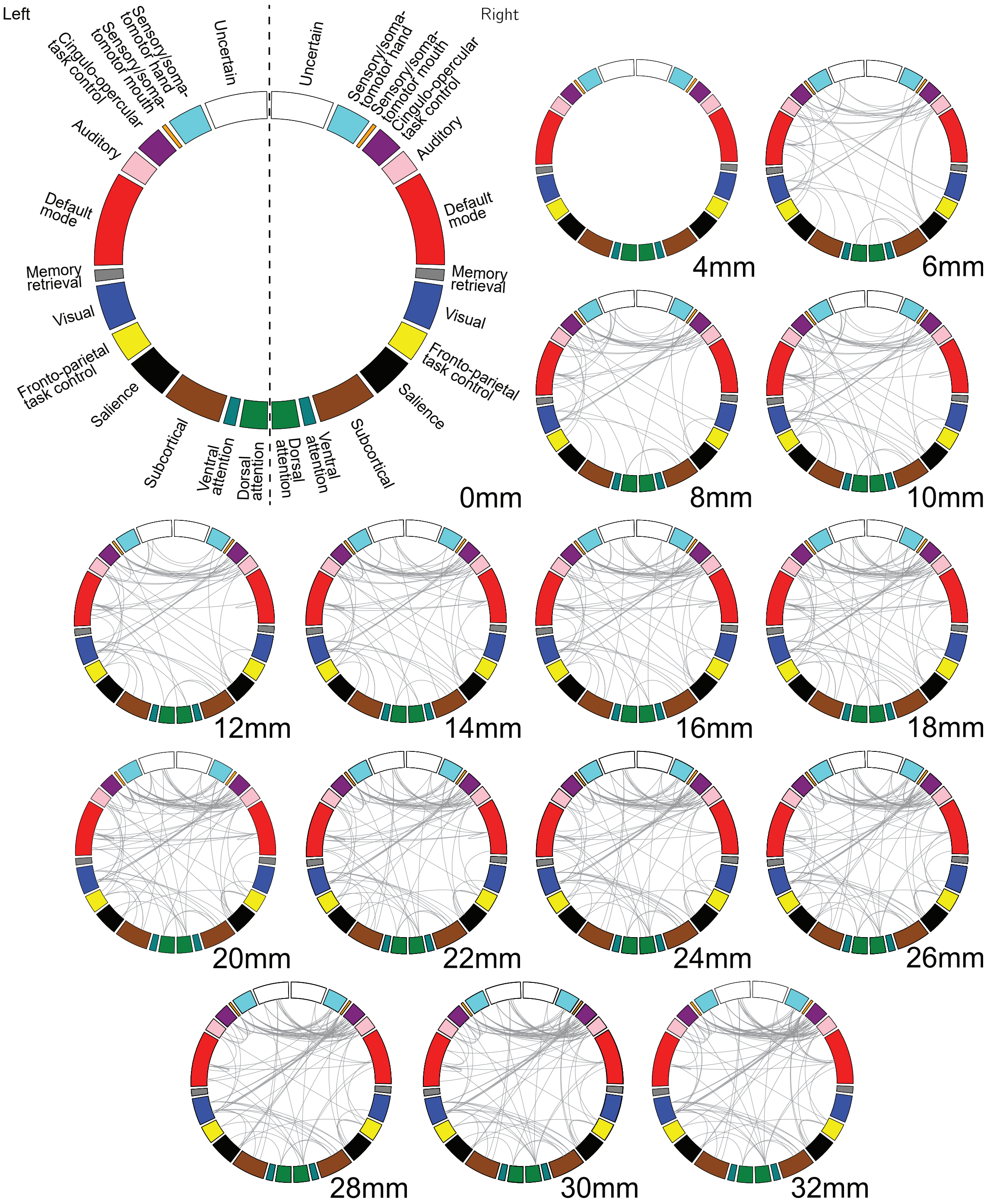

Figure A37: Group-level differences in resting-state functional networks for different smoothing kernels (UCLA dataset, Brainnetome parcellation, adaptive smoothing). The circos plots show the between-groups connectivity differences identified by NBS as described in the Network comparison section of the main article. The nodes are grouped into systems according to Power et al. (2011), colored accordingly, and split in left and right hemispheres. The choice of smoothing kernel changes the detected connectivity differences. No links are detected at FWHM=0 mm and FWHM=4 mm.

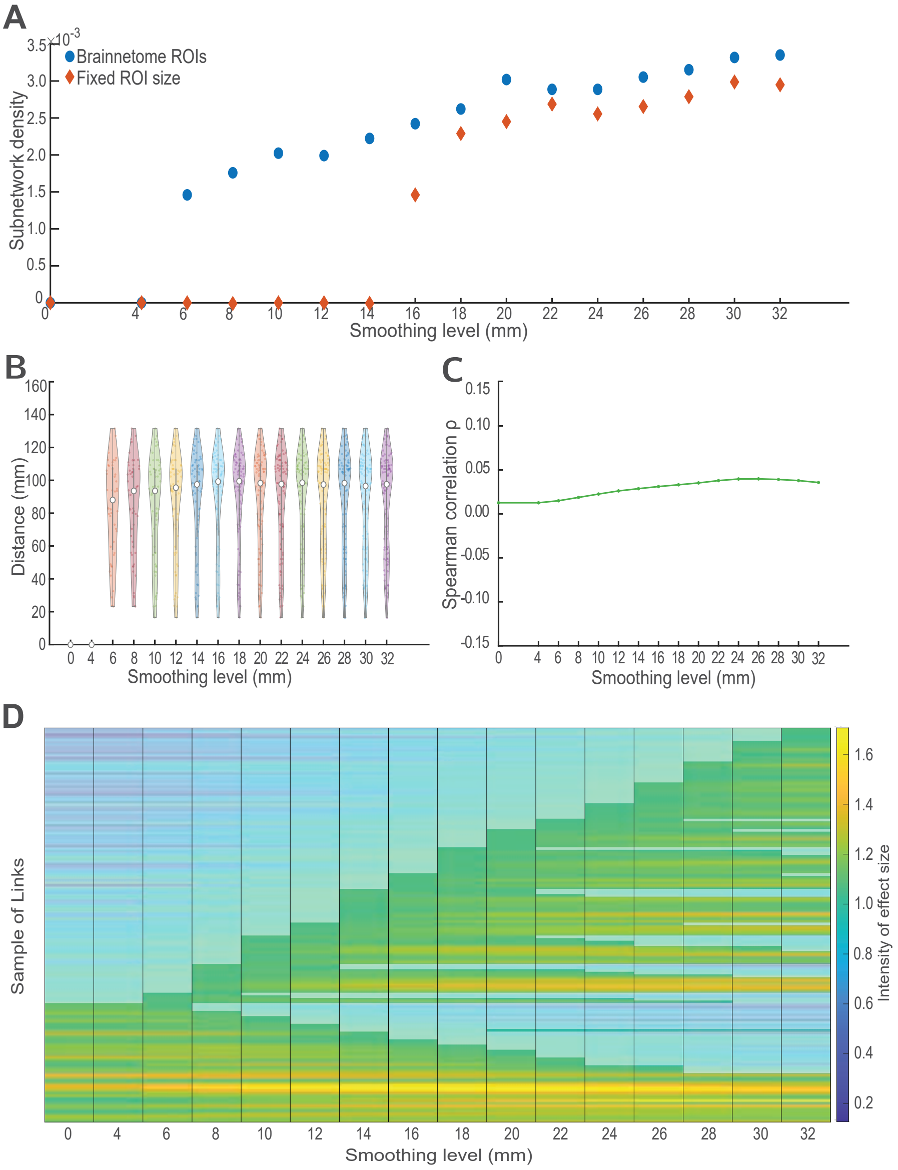

Figure A38: Characteristics of between-group differences according to the smoothing kernel (UCLA dataset, adaptive smoothing). Based on the subnetworks identified by NBS, we see that: (A) The choice of smoothing kernel affects the number of links of the subnetworks and its effects are independent of ROI size. Initially, the number of links in the subnetwork increases with kernel increments. This pattern is present even when the ROIs are artificially constructed as spheres of constant ratios. (B) Spatial smoothing alters the distance profile of detected links. (C) Larger kernels are associated with both, an increase (from 0 to 26 mm), and a decrease (from 28 to 32 mm) in the detection of longer links. A decline in the value of ρ with increasing FWHM highlights a stronger negative correlation between the length of the links and their F-statistic, in other words, using larger kernels will decrease the chances of finding long links. Conversely, an increase in the value of ρ with increasing FWHM highlights a stronger positive correlation between the length of the links and their F-statistic, in other words, using larger kernels will increase the chances of finding long links. (D) Effect sizes of the subnetwork links change depending on the smoothing kernel. The figure shows shows the smoothing kernels on the x-axis, ordered from smallest (0 mm) to largest (32 mm). In the y-axis, the figure shows the links which NBS identified as different between groups at some kernel. The links (y-axis) are organized according to the smallest kernel in which they are detected and the number of smoothing levels in which they appear statistically significant (α<0.05); for example, all links which are significant at 0 mm are shown at the bottom of the plot, then on top of them, we show all links which are significant at 4mm, but are not significant at 0mm. This organization follows until all smoothing levels are shown. The plot highlights those kernels at which the links are found significant. No links are detected at all smoothing levels. Conversely, some links are only observed when a particular kernel is used.

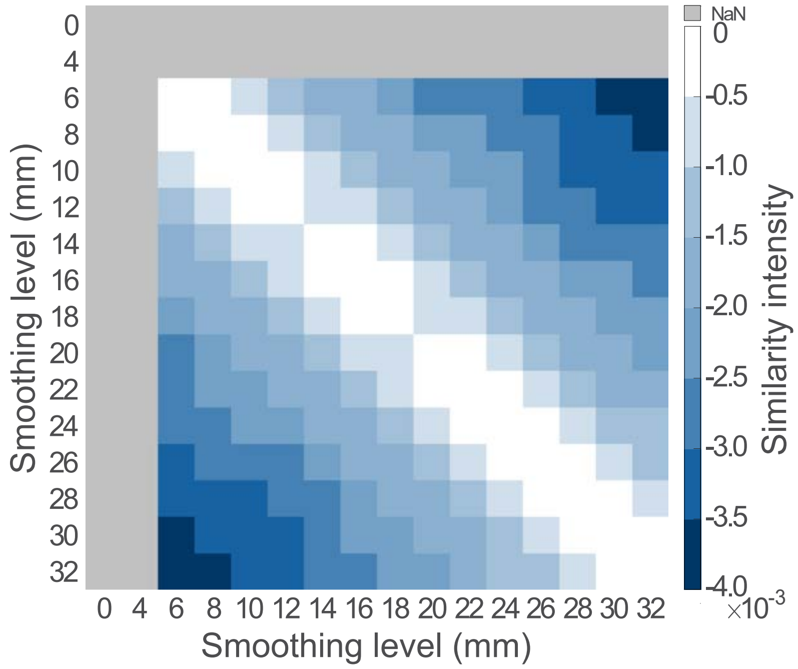

Figure A39: Similarity matrix between the identified subnetwork differences at each smoothing level (UCLA dataset, adaptive smoothing). Brighter colors represent less differences in the structure of the subnetworks. Gray colors show smoothing kernels for which no differences are found. Subnetworks pairs whose values differ in 2 mm show the smallest differences between them (e.g. 6-8 mm, 8-10 mm, etc.).

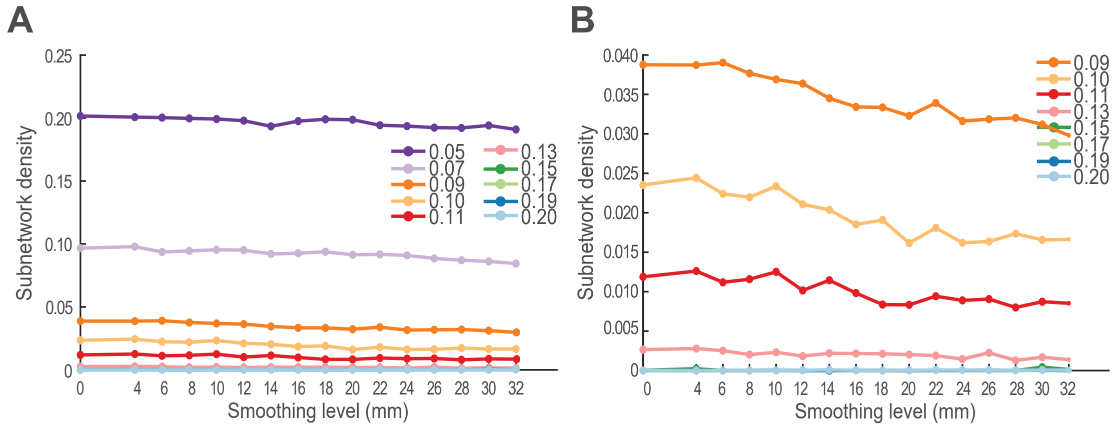

Figure A40: Network density thresholds influence the number of significantly different links comprising the set of between-group differences. We compare the number of links yielded by the permutation tests at 10 different threshold values (UCLA dataset, adaptive smoothing). In general, the number of significantly different links decreases with the increment in the smoothing kernel width for all tested thresholds.

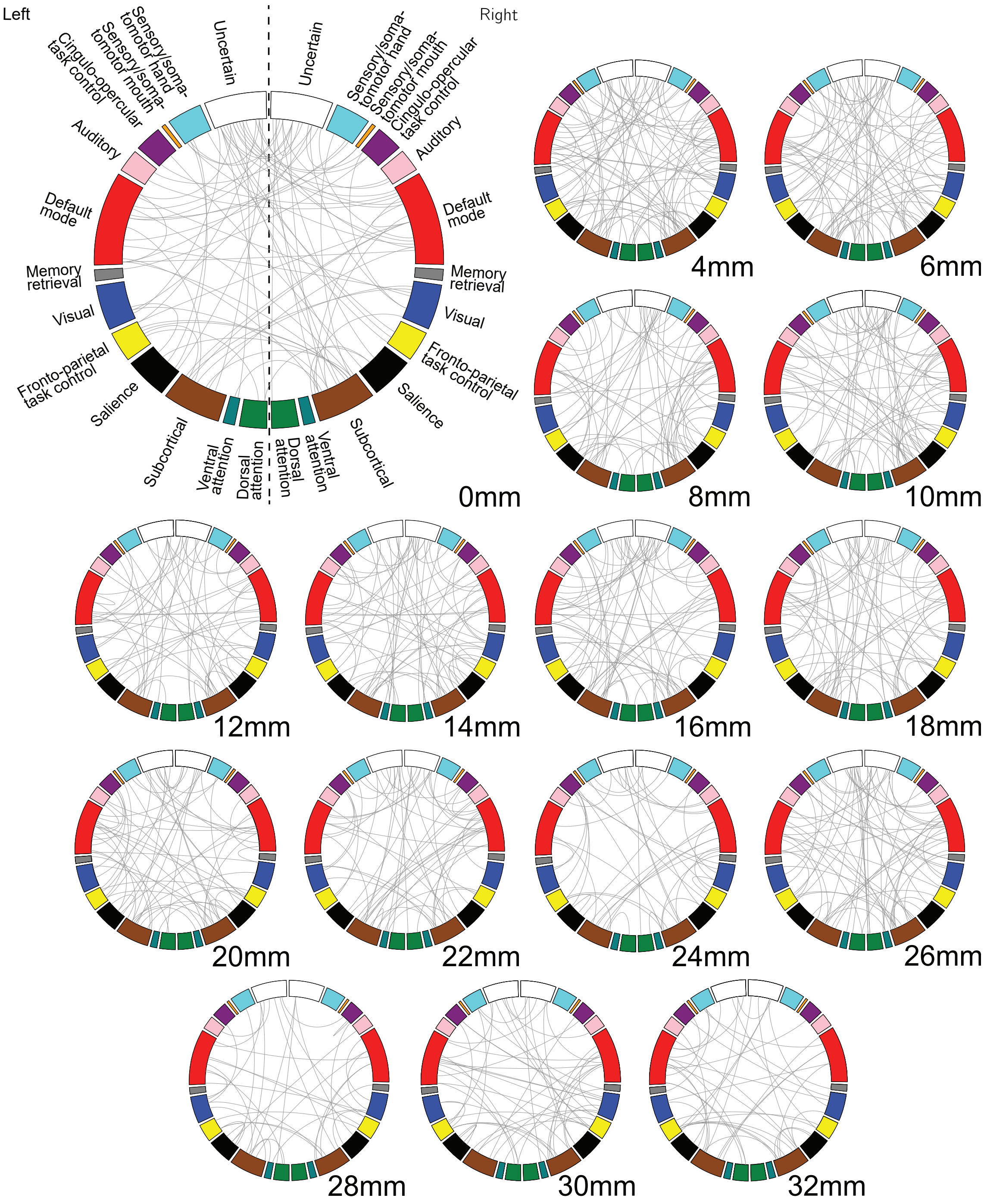

Figure A41: Group-level differences in thresholded, weighted resting-state functional networks for different smoothing kernels (UCLA dataset, Brainnetome parcellation, adaptive smoothing). The circos plots show the between-group connectivity differences identified by permutation tests as described in the Network comparison section of the main article. The nodes are grouped into systems according to Power et al. (2011), colored accordingly, and split in left and right hemispheres. The choice of smoothing kernel changes the detected connectivity differences.

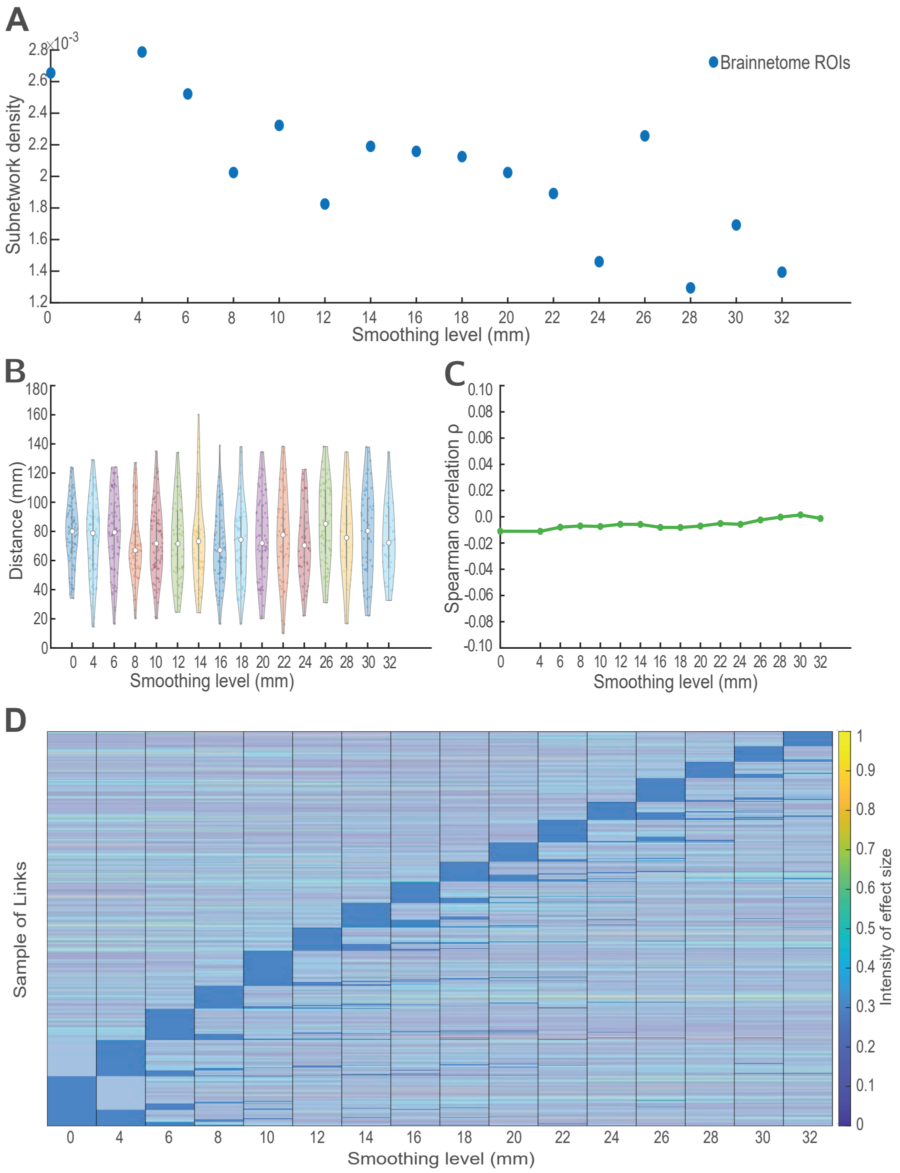

Figure A42: Characteristics of between-group differences according to the smoothing kernel for thresholded, weighted networks (UCLA dataset, Brainnetome parcellation, adaptive smoothing). Based on the network differences identified by the permutation tests, we see that: (A) The choice of smoothing kernel affects the number of significantly different links. In general, the number of significantly different links decreases. (B) Spatial smoothing alters the distance profile of detected links with no clear pattern. (C) No variation is shown between the value of ρ and FWHM, i.e. no relationship is found between the smoothing kernel and the longiture of the signifcant links found. (D) Effect sizes of the significantly different links change depending on the smoothing kernel. The figure shows the smoothing kernels on the x-axis, ordered from smallest (0 mm) to largest (32 mm). In the y-axis, the figure shows the links which permutation tests identified differences between groups at some kernel. The links (y-axis) are organized according to the smallest kernel in which they are detected and the number of smoothing levels in which they appear statistically significant (corrected p-value α<0.05); for example, all links which are significant at 0 mm are shown at the bottom of the plot, then on top of them, we show all links which are significant at 4mm, but are not significant at 0mm. This organization follows until all smoothing levels are shown. The plot highlights those kernels at which the links are found significant. No links are detected at all smoothing levels. Some links are detected at kernels that are not consecutive or at single kernels.

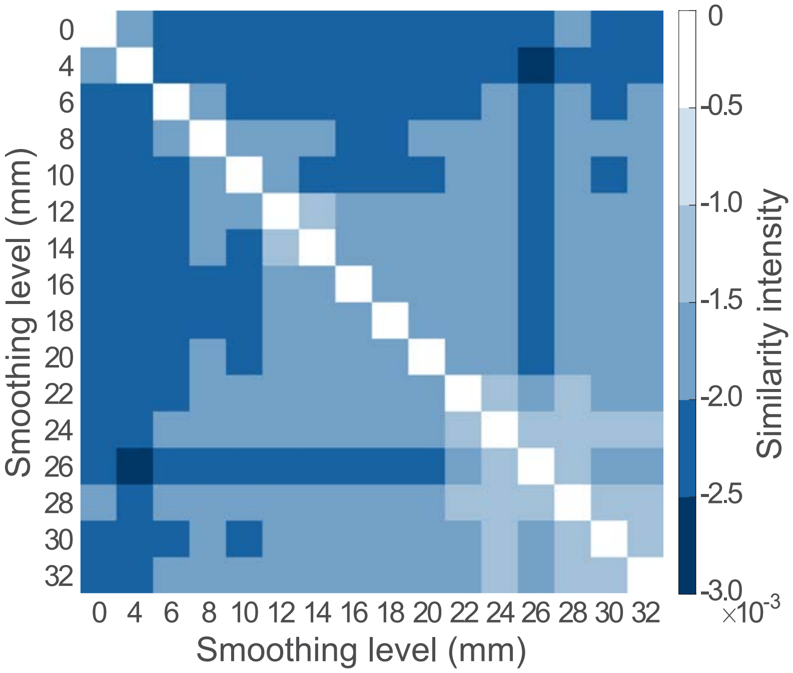

Figure A43: Similarity matrix between the identified subnetwork differences at each smoothing level (UCLA dataset, adaptive smoothing). Brighter colors represent less differences in the structure of the significantly different links. Surprisingly, detected network differences are more similar for larger kernels (FWHM≥20 mm) than for smaller kernel (FWHM≤12 mm) pairs.

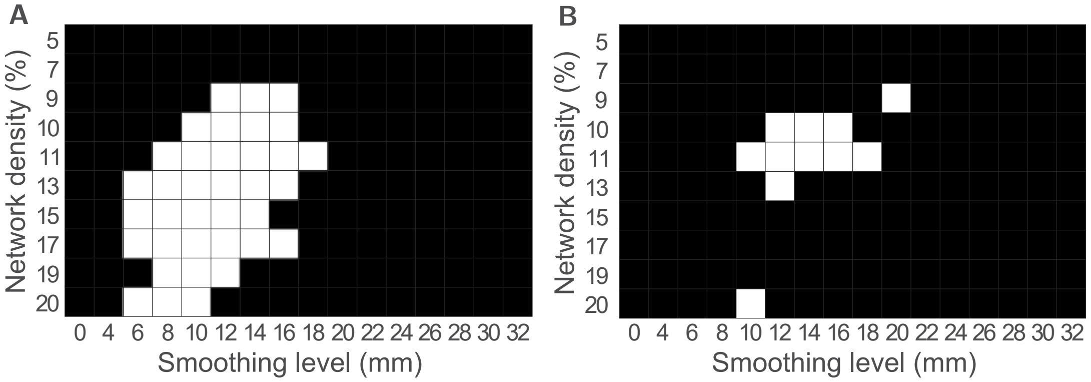

Figure A44: Significant differences are found in the degree of nodes (A) right medial area 8 and (B) left caudal temporal thalamus in the UCLA dataset using data smoothed with adaptive methods. The differences are detected consistently across different network densities and for commonly used kernels. These results are robust for different smoothing levels and thresholds.
